# The burden of ischemic heart disease and the epidemiologic transition in the Eastern Mediterranean Region: 1990–2019

**DOI:** 10.1371/journal.pone.0290286

**Published:** 2023-09-05

**Authors:** Masoumeh Sadeghi, Marjan Jamalian, Kamran Mehrabani-Zeinabad, Karam Turk-Adawi, Jacek Kopec, Wael AlMahmeed, Hanan F. Abdul Rahim, Hasan Ali Farhan, Wagida Anwar, Yosef Manla, Ibtihal Fadhil, Michelle Lui, Hamidreza Roohafza, Sheikh Mohammed Shariful Islam, Kadhim Sulaiman, Nooshin Bazargani, George Saade, Nejat Hassen, Amani Alandejani, Amr Abdin, Saira Bokhari, Gregory A. Roth, Catherine Johnson, Benjamin Stark, Nizal Sarrafzadegan, Ali H. Mokdad

**Affiliations:** 1 Cardiovascular Research Institute, Cardiac Rehabilitation Research Center, Isfahan University of Medical Sciences, Isfahan, Iran; 2 Cardiovascular Research Institute, Hypertension Research Center, Isfahan University of Medical Sciences, Isfahan, Iran; 3 Cardiovascular Research Institute, Pediatric Cardiovascular Research Center, Isfahan University of Medical Sciences, Isfahan, Iran; 4 Department of Public Health, QU-Health, Qatar University, Doha, Qatar; 5 School of Population & Public Health, University of British Columbia, Vancouver, Canada; 6 Arthritis Research Canada, Vancouver, Canada; 7 Heart and Vascular Institute, Cleveland Clinic Abu Dhabi, Abu Dhabi, United Arab Emirates; 8 Department of Public Health, College of Health Sciences, QU Health, Qatar University, Doha, Qatar; 9 Scientific Council of Cardiology, Iraqi Board for Medical Specializations. Baghdad Heart Center, Baghdad, Iraq; 10 Faculty of Medicine, Community Medicine Department, Ain Shams University, Egypt and Armed Forces College of Medicine (AFCM), Cairo, Egypt; 11 Faculty of Medicine, Aleppo University, Aleppo, Syria; 12 Eastern Mediterranean NCD Alliance, Alsafat, Kuwait; 13 Cardiovascular Research Institute, Interventional Cardiology Research Center, Isfahan University of Medical Sciences, Isfahan, Iran; 14 Institute for Physical Activity and Nutrition, Deakin University, Melbourne, Australia; 15 National Heart Center, Royal Hospital, Muscat, Oman; 16 Department of Cardiology, Dubai Hospital, Dubai, UAE; 17 Department of Cardiology, Bellevue Medical Center, Beirut, Lebanon; 18 Syrian Cardiovascular Association, Damascus, Syria; 19 Department of Medicine, Aga Khan University Hospital, Karachi, Pakistan; 20 Institute for Health Metrics and Evaluation, University of Washington, Seattle, United States of America; 21 Department of Health Metrics Sciences, University of Washington, Seattle, United States of America; 22 Cardiovascular Research Institute, Isfahan Cardiovascular Research Center, Isfahan University of Medical Sciences, Isfahan, Iran; Dow University of Health Sciences, PAKISTAN

## Abstract

It has been estimated that in the next decade, IHD prevalence, DALYs and deaths will increase more significantly in EMR than in any other region of the world. This study aims to provide a comprehensive description of the trends in the burden of ischemic heart disease (IHD) across the countries of the Eastern Mediterranean Region (EMR) from 1990 to 2019. Data on IHD prevalence, disability-adjusted life years (DALYs), mortality, DALYs attributable to risk factors, healthcare access and quality index (HAQ), and universal health coverage (UHC) were extracted from the Global Burden of Disease (GBD) database for EMR countries. The data were stratified based on the social demographic index (SDI). Information on cardiac rehabilitation was obtained from publications by the International Council of Cardiovascular Prevention and Rehabilitation (ICCPR), and additional country-specific data were obtained through advanced search methods. Age standardization was performed using the direct method, applying the estimated age structure of the global population from 2019. Uncertainty intervals were calculated through 1000 iterations, and the 2.5th and 97.5th percentiles were derived from these calculations. The age-standardized prevalence of IHD in the EMR increased from 5.0% to 5.5% between 1990 and 2019, while it decreased at the global level. In the EMR, the age-standardized rates of IHD mortality and DALYs decreased by 11.4% and 15.4%, respectively, during the study period, although both rates remained higher than the global rates. The burden of IHD was found to be higher in males compared to females. Bahrain exhibited the highest decrease in age-standardized prevalence (-3.7%), mortality (-65.0%), and DALYs (-69.1%) rates among the EMR countries. Conversely, Oman experienced the highest increase in prevalence (14.5%), while Pakistan had the greatest increase in mortality (30.0%) and DALYs (32.0%) rates. The top three risk factors contributing to IHD DALYs in the EMR in 2019 were high systolic blood pressure, high low-density lipoprotein cholesterol, and particulate matter pollution. The trend analysis over the 29-year period (1990–2019) revealed that high fasting plasma glucose (64.0%) and high body mass index (23.4%) exhibited increasing trends as attributed risk factors for IHD DALYs in the EMR. Our findings indicate an increasing trend in the prevalence of IHD and a decrease in mortality and DALYs in the EMR. These results emphasize the need for well-planned prevention and treatment strategies to address the risk factors associated with IHD. It is crucial for the countries in this region to prioritize the development and implementation of programs focused on health promotion, education, prevention, and medical care.

## Introduction

Ischemic heart disease (IHD) stands as a major contributor to the global burden of disease, with severe implications for human health worldwide [1]. Over the past decades, IHD has emerged as the leading cause of mortality in both men and women across various regions [2]. Notably, the burden of disability-adjusted life years (DALYs) attributed to IHD tends to be considerably higher among men compared to women in most parts of the world [3]. Individuals diagnosed with IHD necessitate specialized and costly services that require prompt interventions to avert potential complications [4].

The Eastern Mediterranean Region (EMR) exhibits a wide range of cultural, linguistic, political, social, historical, and economic diversity [5]. In 2019, the estimated healthy life expectancy in the EMR was 60.2 years for males and 60.7 years for females. The average health expenditure in the region was -$695.3, ranging from -$36 to -$2106 [6]. EMR countries have been categorized into five social demographic index (SDI) levels by the Global Burden of Disease (GBD), namely high (three countries), high middle (six countries), middle (five countries), low middle (three countries), and low (four countries) [7].

Projections indicate that in the coming decade, the EMR will experience a more significant increase in IHD prevalence, DALYs, and deaths compared to other regions worldwide [8]. The rising prevalence of IHD in EMR countries may reflect the inadequate healthcare capacity and resources available to address the disease’s risk factors. Notable attributable risk factors contributing to IHD DALYs in the EMR include behavioral factors such as an unhealthy lifestyle (poor diet, low physical activity, and smoking habits), metabolic factors (hypertension, diabetes, and hypercholesterolemia), and environmental factors (e.g., air pollution) [8–10]. Moreover, the implementation of preventive strategies and patient follow-up varies significantly among EMR countries, as evident in the availability of cardiac rehabilitation (CR). CR, a guideline-recommended secondary preventive strategy, plays a crucial role in managing IHD risk factors and reducing mortality rates by 20% [9].

Understanding the epidemiological transition and comprehending the burden of IHD, as well as the availability of cardiac rehabilitation services in the EMR, hold paramount importance for informed decision-making and the development of future strategies for IHD prevention and treatment [10,11]. This study aims to present the trends in the burden of IHD, including prevalence, DALYs, mortality, and the availability of cardiac rehabilitation services, across EMR countries. Additionally, we provide an analysis of the burden measures categorized by sex and SDI groups for EMR countries between 1990 and 2019.

## Materials and methods

This epidemiological study utilized data on IHD in EMR countries from 1990 to 2019. The data were collected through an online query tool, namely the Institute for Health Metrics and Evaluation (IHME, Global Burden of Disease Compare), and advanced searches in databases such as PubMed, Google Scholar, and EMR countries’ websites within the specified time period.

The EMR consists of 22 countries, encompassing a population of 583 million individuals [12]. For this study, data from Palestine were unavailable and therefore not included. The countries were classified into five socio-demographic index (SDI) categories: high-SDI (Kuwait, United Arab Emirates, Qatar), high-middle SDI (Libya, Jordan, Saudi Arabia, Lebanon, Bahrain, Oman), middle SDI (Tunisia, Islamic Republic of Iran, Iraq, Syrian Arab Republic, Egypt), low-middle SDI (Djibouti, Morocco, Sudan), and low SDI (Somalia, Pakistan, Yemen, Afghanistan) [7].

Data were extracted from the IHME(http://ghdx.healthdata.org/gbd-results-tool), including age-standardized IHD prevalence, death rates, DALYs rates, and DALYs attributable to risk factors (behavioral, environmental, and metabolic), stratified by sex and SDI status. Healthcare access and quality index (HAQ) data and universal healthcare coverage (UHC) stratified by SDI status were also extracted [8]. UHC refers to the average coverage of necessary services, including non-communicable diseases, infectious diseases, reproductive, maternal, newborn, and child health, as well as service capacity and access [13]. HAQ is a measure of health access and quality of care derived from death rates associated with preventable causes [14].

Additionally, information on the availability of cardiac rehabilitation (CR) was obtained from publications by the International Council of Cardiovascular Prevention and Rehabilitation (ICCPR) from 1990 to 2019, with a focus on the CR survey conducted in 2015. CR capacity was calculated by multiplying the median number of patients a program could serve per year (from the survey) by the number of programs in each country.

Advanced search methods were employed to gather data on angiography, primary percutaneous coronary intervention (PPCI), and preventive community-based interventions (PCBI) [15] from databases such as PubMed, Google Scholar, and EMR countries’ websites. It is worth noting that EMR countries have varying infrastructure, equipment, and facilities in primary healthcare, hospitals, and the private sector [16].

The Socio-Demographic Index (SDI), a composite indicator of development status strongly correlated with health outcomes, was used to categorize countries based on their development continuum in 2019 [17]. EMR countries were grouped into five categories based on SDI quintiles: low SDI (Afghanistan and Yemen), low-middle SDI (Morocco, Palestine, and Sudan), middle SDI (Algeria, Egypt, Iran (Islamic Republic of), Iraq, Syrian Arab Republic, and Tunisia), high-middle SDI (Bahrain, Jordan, Lebanon, Libya, Oman, Saudi Arabia, and Turkey), and high SDI (Kuwait, Qatar, and United Arab Emirates). The Healthcare Quality and Access (HAQ) index, constructed using risk-standardized cause-specific death rates, facilitated comparisons over time and geography [18]. Age-standardized rates of IHD deaths and DALYs by country, gender, and year were obtained from GBD 2019 using the GBD results tool [19].

Age standardization was performed using the direct method, applying the estimated age structure of the global population from 2019 [20]. Uncertainty intervals were calculated through 1000 iterations, and the 2.5th and 97.5th percentiles were derived from these calculations [21]. Detailed methodology for calculating burden in GBD papers published by IHME groups. For analysis of the burden attributed risk, we used ready-made information by GBD and did not estimate it. All figures were generated using R software, version 4.2.2.

## Results

In this study, the age-standardized prevalence of IHD in the EMR exhibited a 7.06% increase from 1990 to 2019, while the global level experienced a decrease of 3.44% in age-standardized prevalence (S1 File). Analyzing the 29-year trend, Bahrain demonstrated the highest decrease (-3.72%), whereas Oman showed the highest increase (14.40%) in age-standardized prevalence (Table 1, Fig 2). Furthermore, the prevalence of IHD was generally higher in males compared to females, and older patients exhibited higher prevalence within the EMR (S2–S4 Files).

**Table 1:** Comprasion of age-standardized IHD prevalence (per 100,000) in 1990, 2005 and 2019, and their relative percentage change by SDI level and EMR countries.

SDI	Countries	Prevalence (%) (95%UI) *			%Δ ($\frac{x_{i+1}-x_{i}}{x_{i}}$)		
		1990	2005	2019	1990–2005	2005–2019	1990–2019
**Total**	Global	2.62 (2.35–2.91)	2.55 (2.32–2.81)	2.53 (2.28–2.81)	-2.67	-0.78	-3.44
	EMR	4.96 (4.56–5.38)	5.21 (4.86–5.59)	5.31 (4.89–5.75)	5.04	1.92	7.056
**High**	Kuwait	5.75 (5.32–6.21)	6.07 (5.68–6.50)	5.98 (5.53–6.45)	5.56	-1.483	4.00
	United Arab Emirates	5.05 (4.68–5.45)	5.19 (4.87–5.51)	5.52 (5.11–5.96)	2.77	6.35	9.31
	Qatar	5.00 (4.62–5.41)	5.17 (4.84–5.53)	5.17 (4.78–5.58)	3.40	0	3.40
**High middle**	Libya	4.72 (4.38–5.10)	5.05 (4.73–5.40)	5.15 (4.77–5.99)	6.99	1.98	9.11
	Jordan	5.50 (5.08–5.95)	5.49 (5.14–5.90)	5.42 (5.02–5.87)	-0.18	-1.275	-1.45
	Saudi Arabia	4.81 (4.45–5.17)	5.33 (5.00–5.69)	5.48 (5.08–5.91)	10.81	2.81	13.93
	Lebanon	5.07 (4.71–5.45)	5.02 (4.71–5.35)	5.25 (4.88–5.66)	-0.99	4.58	3.55
	Bahrain	5.65 (5.23–6.11)	5.31 (4.97–5.68)	5.44 (5.03–5.90)	-6.02	2.44	-3.72
	Oman	5.07 (4.69–5.48)	5.53 (5.19–5.93)	5.80 (5.38–6.23)	9.07	4.88	14.40
**Middle**	Tunisia	4.59 (4.25–4.94)	4.70 (4.40–5.02)	4.74 (4.40–5.11)	2.40	0.85	3.27
	Iran (Islamic Republic of)	6.55 (5.96–7.19)	6.65 (6.14–7.20)	6.50 (5.91–7.15)	1.53	-2.26	-0.76
	Iraq	5.83 (5.39–6.29)	5.65 (5.31–6.05)	5.66 (5.23–6.12)	-3.09	0.176	-2.92
	Syrian Arab Republic	5.09 (4.73–5.46)	5.31 (4.99–5.66)	5.40 (5.03–5.80)	4.32	1.69	6.09
	Egypt	5.58 (5.22–6.00)	5.61 (5.31–5.96)	5.89 (5.50–6.31)	0.54	4.99	5.56
**Low middle**	Djibouti	2.12 (1.89–2.40)	2.29 (2.05–2.58)	2.37 (2.11–2.68)	8.02	3.49	11.79
	Morocco	5.51 (5.09–5.94)	5.60 (5.23–6.01)	5.47 (5.06–5.93)	1.63	-2.32	-0.73
	Sudan	5.04 (4.69–5.42)	5.16 (4.84–5.52)	5.30 (4.90–5.70)	2.38	2.71	5.16
**Low**	Somalia	1.84 (1.65–2.07)	1.88 (1.68–2.12)	1.89 (1.68–2.13)	2.17	0.53	2.72
	Pakistan	3.85 (3.45–4.28)	4.17 (3.77–4.62)	4.28 (3.83–4.76)	8.31	2.63	11.17
	Yemen	4.82 (4.48–5.18)	4.88 (4.57–5.21)	5.00 (4.64–5.39)	1.245	2.46	3.73
	Afghanistan	5.45 (5.06–5.86)	5.33 (5.00–5.72)	5.40 (5.00–5.84)	-2.20	1.31	-0.92

*95% uncertainty intervals (UI) gathered from GBD website.

**Fig 1:**
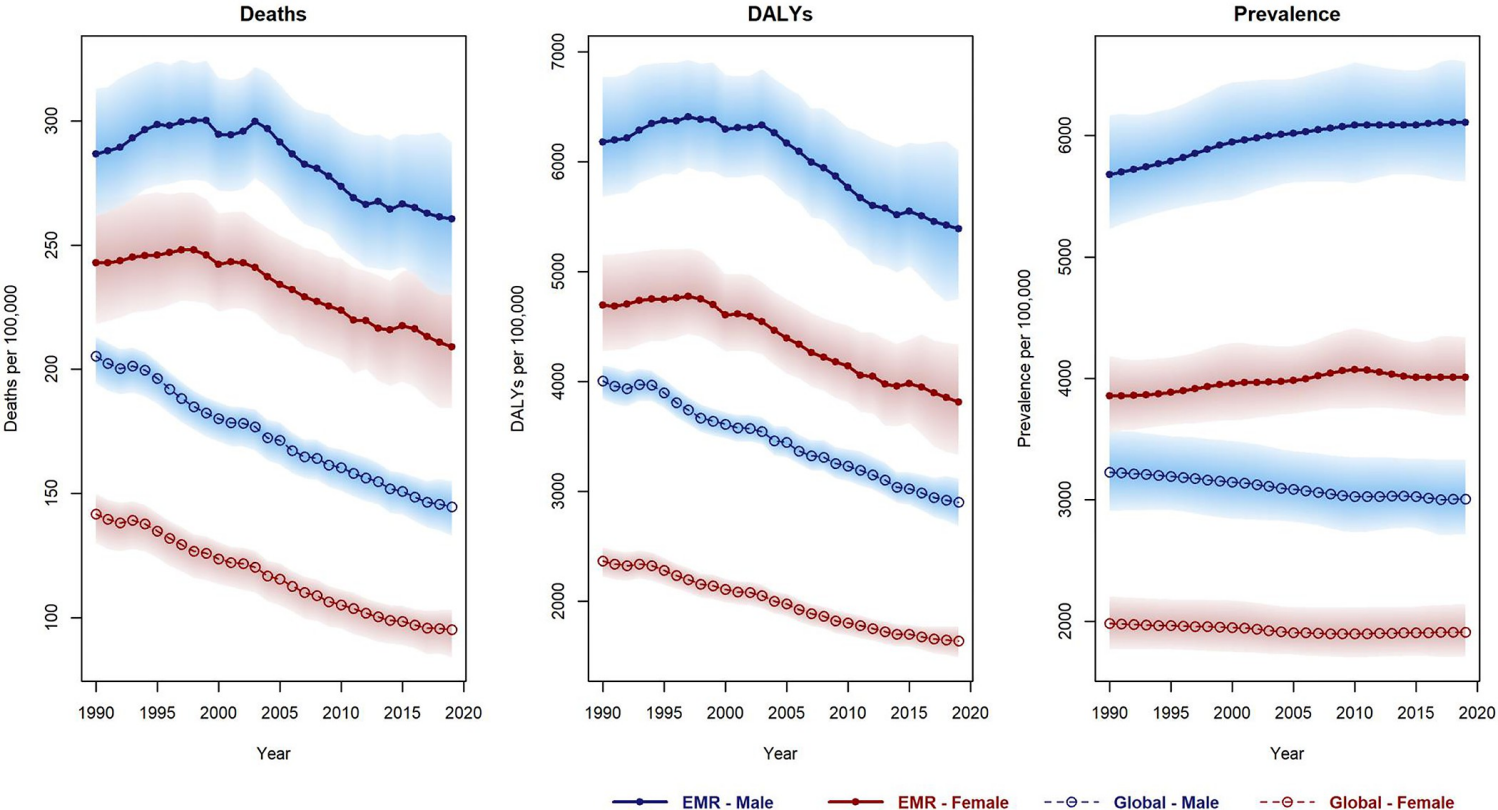
Trend of age-standardized deaths rate, disability-adjusted life years (DALYs) rate and prevalence of IHD for males and females during 1990 to 2019 worldwide and in EMR.

**Fig 2:**
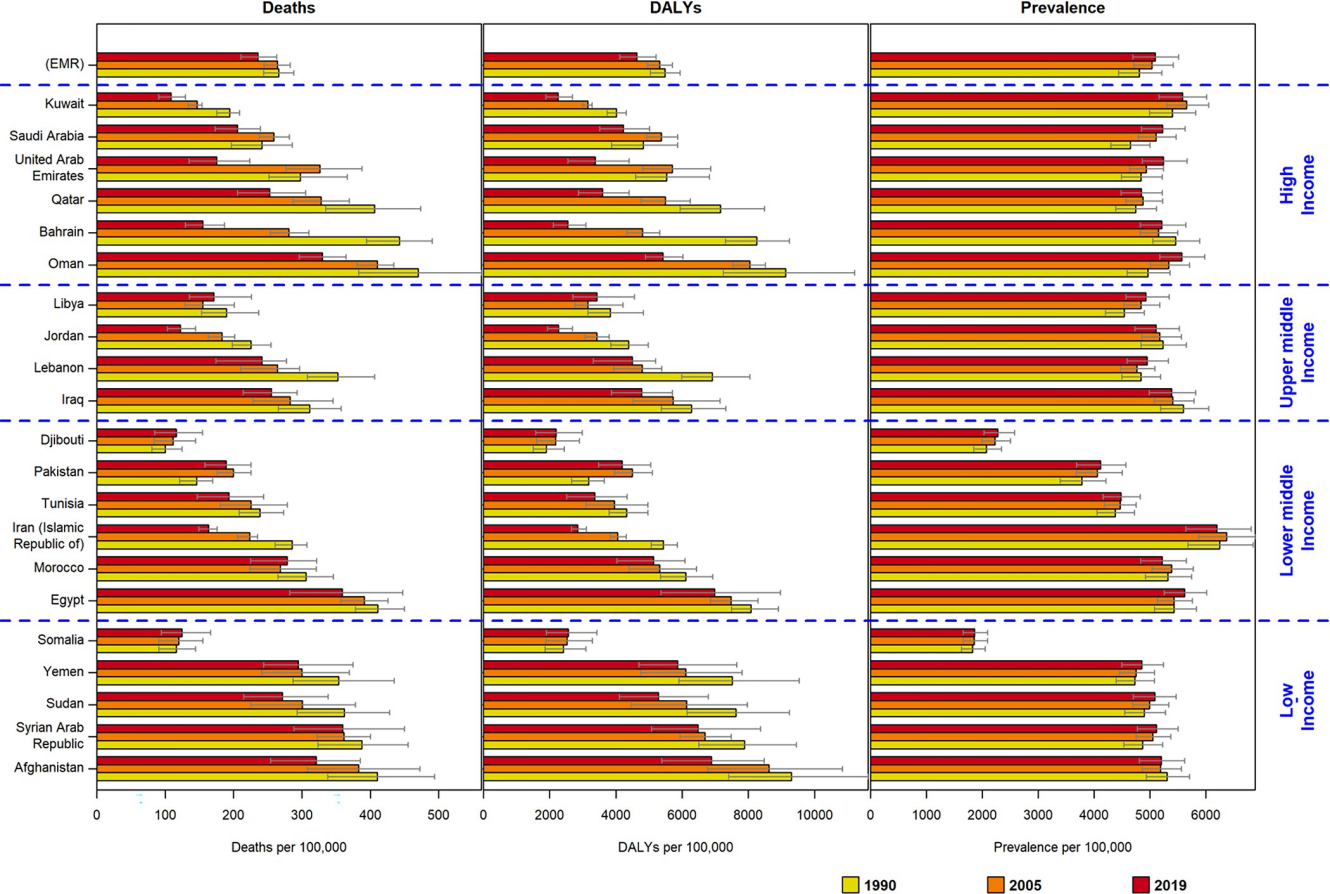
Comparison of age-standardized prevalence, death and disability-adjusted life years (DALYs) rate for IHD (per 100,000) in 1990,2005 and 2019 by SDI level and geographical area.

During the study period, there was a decrease of 11.39% and 15.36% in age-standardized death and DALYs rates for IHD in the EMR, respectively (Tables 2 and 3). In 2019, the age-standardized death and DALYs rates were higher among males and older age patients within the EMR (S6–S9 Files). Analyzing the 29-year trend, Bahrain exhibited the highest decrease (-69.95% for death rate, -69.08% for DALYs rate), while Pakistan showed the highest increase (29.62% for death rate, 31.93% for DALYs rate) (Tables 2 and 3 / Figs 1 and 2). Detailed information on age-standardized death rates for males and females categorized by SDI status and EMR countries is provided in S8 and S9 Files. Older patients within the EMR experienced higher IHD death and DALY rates (S2 File).

**Table 2:** Comparison of age-standardized death rate of IHD (per 100,000) in 1990, 2005 and 2019, and their relative percentage change by SDI level and EMR countries.

SDI	Countries	death (%) (95%UI) *			%Δ ($\frac{x_{i+1}-x_{i}}{x_{i}}$)		
		1990	2005	2019	1990–2005	2005–2019	1990–2019
**Total**	Global	170.45 (159.61–176.94)	141.23 (131.93–147.32)	117.95 (107.83–125.92)	-17.14	-16.48	-30.80
	EMR	266.15 (243.83–287.97)	263.91 (244.39–282.68)	235.83 (210.43–263.11)	-0.84	-10.64	-11.39
**High**	Kuwait	194.57 (175.56–208.87)	146.41 (133.73–153.86)	108.53 (90.73–129.2)	-24.75	-25.87	-44.22
	United Arab Emirates	297.53 (251.6–366.43)	326.04 (276.62–387.52)	175.40 (134.75–223.39)	9.58	-46.20	-41.05
	Qatar	405.98 (334.4–473.78)	327.93 (286.74–368.86)	252.99 (205.75–305.43)	-19.23	-22.85	-37.68
**High middle**	Libya	189.88 (153.37–236.74)	154.84 (128.78–201.14)	171.30 (135.45–226.05)	-18.45	10.63	-9.79
	Jordan	225.39 (197.81–254.57)	182.76 (162.68–201.34)	121.92 (103.15–144.05)	-18.91	-33.29	-45.91
	Saudi Arabia	241.31 (196.57–285.82)	259.05 (237.54–281.32)	205.60 (172.88–238.97)	7.35	-20.63	-14.80
	Lebanon	352.64 (307.84–405.86)	264.43 (210.2–296.66)	241.23 (174.11–277.12)	-25.01	-8.77	-31.59
	Bahrain	442.46 (394.45–490.13)	280.79 (253.64–310.28)	155.09 (129.29–186.67)	-36.54	-44.77	-64.95
	Oman	470.15 (382.97–562.72)	410.40 (380.67–434.34)	329.85 (296.03–364.09)	-12.71	-19.63	-29.84
**Middle**	Tunisia	238.79 (208.11–273.08)	225.69 (180.71–278.33)	193.45 (146.86–244.04)	-5.49	-14.29	-18.99
	Iran (Islamic Republic of)	285.68 (260.83–307.09)	223.38 (205.52–235.21)	163.56 (148.98–176.17)	-21.81	-26.78	-42.75
	Iraq	311.21 (265.52–357.49)	282.37 (228.59–345.26)	255.40 (214.11–292.61)	-9.27	-9.55	-17.93
	Syrian Arab Republic	387.72 (323.52–455.31)	361.50 (322.04–399.96)	359.72 (288.25–449.75)	-6.76	-0.49	-16.65
	Egypt	410.76 (377.9–449.55)	391.00 (356.68–425.62)	359.27 (281.82–447.03)	-4.81	-8.12	-12.54
**Low middle**	Djibouti	99.93 (80.38–124.65)	111.4 (83.64–144.14)	115.99 (84.39–154.53)	11.48	4.12	16.07
	Morocco	306.23 (264.97–346.06)	268.38 (223.51–321.12)	278.54 (224.66–321.41)	-12.36	3.79	-9.04
	Sudan	361.78 (292.57–428.25)	300.27 (224.83–377.85)	271.47 (214.92–338.38)	-17.00	-9.59	-7.22
**Low**	Somalia	116.20 (90.63–144.35)	119.84 (90.71–155.18)	124.60 (94.03–166.56)	3.13	3.97	7.23
	Pakistan	146 (121.02–169.53)	199.52 (175.96–225.5)	189.25 (158.03–225.54)	36.66	-5.15	29.62
	Yemen	353.65 (286.77–433.72)	300.18 (240.83–369.32)	294.76 (243.73–374.52)	-15.12	-1.81	-30.80
	Afghanistan	410.08 (337.36–493.65)	382.71 (307.8–472.38)	320.87 (253.97–385.29)	-6.67	-16.16	7.23

^*^95% uncertainty intervals (UI) gathered from GBD website.

**Table 3:** Comparison age-standardized of disability-adjusted life years (DALYs) rate of IHD (per 100,000) in 1990, 2005 and 2019, and their relative percentage change by SDI level and EMR countries.

SDI	Countries	DALY (%) (95%UI) *			%Δ ($\frac{x_{i+1}-x_{i}}{x_{i}}$)		
		1990	2005	2019	1990–2005	2005–2019	1990–2019
**Total**	Global	3,143.28(3,012.81–3,257.17)	2,681.06(2,571.04–2,788.57)	2,243.54(2,098.7–2,385.01)	-14.7	-16.32	-28.62
	EMR	5,477.18(5,043.35–5,933.13)	5,317.48(4,958.33–5,700.12)	4,635.68(4,111.93–5,215.68)	-2.91	-12.82	-15.36
**High**	Kuwait	4,013.98 (3,729.8–4,309.17)	3,156.76(2,994.85–3,281.76)	2,252.19(1,881.58–2,687.81)	-21.36	-28.65	-43.89
	United Arab Emirates	5,526.74 (4,596.73–6,821.42)	5,703.27(4,809.05–6,862.87)	3,370.13(2,554.97–4,399.53)	3.19	-40.91	-39.02
	Qatar	7,161.85 (5,933.31–8,494.79)	5,489.92(4,751.15–6,247.38)	3,597.19 (2,863.86–4,394.89)	-23.34	-34.48	-49.78
**High middle**	Libya	3,831.86(3,154.32–4,834.38)	3,151.61(2,756.68–4,207.63)	3,428.97(2,699.3–4,556.74)	-17.75	8.80	-10.51
	Jordan	4,382.81 (3,842.32–4,982.28)	3,420.09(3,064.91–3,790.89)	2,265.22(1,931.87–2,687.2)	-21.96	-33.77	-48.32
	Saudi Arabia	4,830.87(3,863.07–5,859.23)	5,368.59(4,933.61–5,864.84)	4,221.82 (3,508.4–5,008.79)	11.13	-21.36	-12.61
	Lebanon	6,914.61 (5,990.59–8,046.14)	4,798.31(3,931.97–5,379.76)	4,491.97(3,316.33–5,196.53)	-30.61	-6.38	-35.04
	Bahrain	8,254.04 (7,309.32–9,242.96)	4,801.56(4,338.42–5,327.85)	2,552.30(2,101.25–3,090.38)	-41.83	-46.84	-69.08
	Oman	9,125.88 (7,242.9–11,207.84)	8,545.45(7,521.95–8,510.17)	5,417.45(4,890.81–6,023.60)	-6.36	-36.60	-40.64
**Middle**	Tunisia	4,324.18(3,799.93–4,963.54)	3,951.27(3,096.41–4,965.18)	3,357.38(2,517.60–4,339.07)	-8.62	-15.03	-22.36
	Iran (Islamic Republic of)	5,437.6(5,060.86–5,851.02)	4,053.22(3,832.64–4,312.97)	2,842.75(2,657–3,103.12)	-25.46	-29.86	-47.72
	Iraq	6,281.45 (5,368.85–7,318.89)	5,723.26(4,523.97–7,147.77)	4,775.69 (1,931.87–2,687.2)	-8.89	-16.56	-23.97
	Syrian Arab Republic	7,885.82 (6,504.02–9,453.26)	6,688.44(5,933.23–7,472.83)	6,479.76(5,077.38–8,364.95)	-15.18	-3.12	-17.83
	Egypt	8,085.34 (7,486.43–8,913.91)	7,481.77(6,849.42–8,289.95)	6,986.42(5,361.90–8,973.94)	-7.46	-6.62	-13.59
**Low middle**	Djibouti	1,895.45(1,503.3–2,433.55)	2,176.62(1,602.59–2,896.5)	2,189.19(1,573.55–2,975.72)	14.83	0.58	15.50
	Morocco	6,114.72(5,347.42–6,928.7)	5,317.03 (4,397.09–6,429.95)	5,131.83(4,026.02–6,088.91)	-13.04	-3.48	-16.07
	Sudan	7,625.12(6,145.22–9,237.55)	6,131.44(4,460.27–7,968.63)	5,290.1(4,103.35–6,787.13)	-19.59	-13.72	-30.62
**Low**	Somalia	2,416.05 (1,854.11–3,094.57)	2,522.94(1,889.54–3,291.95)	2,566.51(1,896.94–3,429.77)	4.42	1.73	6.23
	Pakistan	3,178.83(2,659.8–3,642.27)	4,495.11(3,957.73–5,097.18)	4,193.96(3,477.81–5,051.64)	41.41	-6.70	31.93
	Yemen	7,520.41(5,906.62–9,540.02)	6,105.79(4,743.54–7,811.11)	5,859.78(4,694.65–7,645.44)	-18.81	-4.029	-22.08
	Afghanistan	9,297.15(7,407.11–11,625.33)	8,629.81(6,758.08–10,847)	6,883.5(5,382.21–8,475.38)	-7.18	-20.24	-25.96

^*^95% uncertainty intervals (UI) gathered from GBD website.

**Fig 3:**
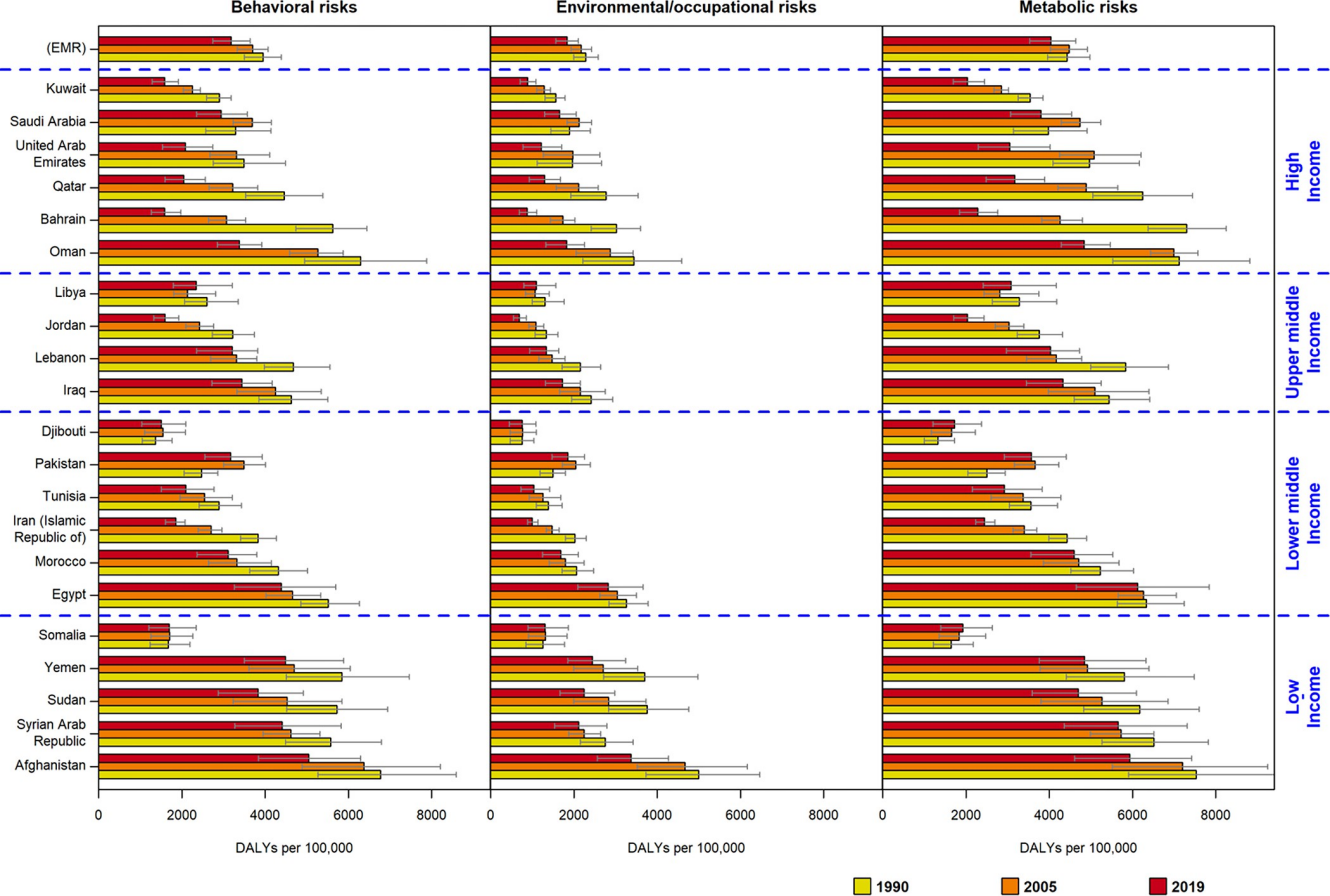
Comparison of age-standardized attributed risk factor to disability-adjusted life years (DALYs) rate (per 100,000) for behavioral, metabolic and environmental risk factors by SDI level and geographical area.

Examining the 29-year change percentage of DALYs attributable to risk factors, Bahrain exhibited the highest decrease in metabolic (-68.78%), behavioral (-71.76%), and environmental (-70.99%) factors. Conversely, Pakistan demonstrated the highest increase in the 29-year change percentage for metabolic (42.54%), behavioral (28.26%), and environmental (24.04%) factors (S9 File, Fig 3).

Among the behavioral risk factors, Pakistan displayed the highest increase in the trend of dietary risk and low physical activity during the period of 1990–2019 (30.96% and 32.76%, respectively), while Djibouti showed the highest increase in the trend of tobacco use (11.78%). Bahrain exhibited the highest decrease in the trend of dietary risk, low physical activity, and tobacco use (-71.70%, -64.85%, and -76.90%, respectively) (S10 File).

The top three risk factors contributing to attributable IHD DALY rates in the EMR were high systolic blood pressure, high LDL-cholesterol, and particulate matter air pollution during the study years. The relative change in the 29-year trend of attributed risk factors for IHD DALYs in the EMR (1990–2019) revealed an increasing trend for high fasting plasma glucose (64.03%) and high BMI (23.39%) (S11 File).

Analyzing the relationship between HAQ index and DALYs (1990–2015), it was observed that Somalia, Djibouti, and Pakistan experienced an increase in DALYs, likely associated with an increase in HAQ. However, Bahrain showed a decrease in DALYs, possibly indicating an increase in HAQ (S12 File). It is important to note that considering the lag time between HAQ and burden indicators, these findings cannot be confidently concluded.

The trend analysis of the percentage of relative change in UHC (1990–2019) revealed that Pakistan had the lowest change (7.78%), while Sudan had the highest change (88.69%). It is crucial to consider the lag time in this aspect as well. Angiography and angioplasty facilities were available in EMR countries, and primary preventive community-based intervention programs were prevalent in the majority of countries in the region. Regarding cardiac rehabilitation (CR) in the EMR, Iran (Islamic Republic of) had the highest number of CR programs (Table 4).

**Table 4:** Information on health services and cardiac rehabilitation (CR) availability in the EMR countries by SDI level.

SDI level	Countries	UHC^¥^			PPCI^+^	PCBI*	Angiography and Angioplasty**	CR capacity needed (2017)	CR program number
		1990	2019	%Δ ($\frac{x_{i+1}-x_{i}}{x_{i}}$) 1990–2019					
**Total**	EMR	45.35	59.84	31.95	Π	Π	Π	69,487	1
**High**	Kuwait	65.25	81.83	25.41	Π	Π	Π	7222	1
	United Arab Emirates	55.06	63.36	15.08	Π	Π	Π	21,639	1
	Qatar	59.88	80.40	34.27	Π		Π	6811	1
**High middle**	Libya	57.77	66.33	14.82	Π	Π	Π	NA	NA
	Jordan	52.99	69.97	32.03	Π	Π	Π	NA	NA
	Saudi Arabia	50.21	64.19	27.86	Π	Π	Π	82,264	1
	Lebanon	54.24	74.53	37.40	Π	Π	Π	27,333	1
	Bahrain	54.84	70.58	28.69	Π	-	Π	3342	1
	Oman	53.58	71.22	32.94	Π	Π	Π	NA	NA
**Middle**	Tunisia	55.86	68.11	21.92	Π	Π	Π	50,067	1
	Iran (Islamic Republic of)	52.53	69.51	32.33	Π	Π	Π	219,007	34
	Iraq	45.37	57.72	27.23	Π	NA	Π	NA	NA
	Syrian Arab Republic	45.41	57.56	26.77	Π	Π	Π	NA	NA
	Egypt	38.16	54.80	43.58	Π	Π	Π	369,288	2
**Low middle**	Djibouti	38.94	45.29	16.30	Π		Π	NA	NA
	Morocco	41.01	58.03	41.50	Π		Π	155,842	1
	Sudan	27.47	51.83	88.69	Π	Π	Π	NA	NA
**Low**	Somalia	15.26	23.94	56.84	Π	Π	Π	NA	NA
	Pakistan	36.34	39.17	7.78	Π	Π	Π	616,146	4
	Yemen	30.24	49.05	62.16	Π	NA	Π	NA	NA
	Afghanistan	22.01	39.29	78.51	Π	Π	Π	88,906	1

^¥^ UHC: Universal health coverage index

^+^PPCI: Primary Percutaneous Coronary Intervention.https://www.ncbi.nlm.nih.gov/pmc/articles/PMC3129088/

*PCBI: Preventive Community-based Intervention.

** based search of countries.

CR: Cardiac rehabilitation (45).

NA: None-available and missing.

## Discussion

Our study revealed several key findings regarding the burden of ischemic heart disease (IHD) in the Eastern Mediterranean Region (EMR) from 1990 to 2019. While there was a decrease in age-adjusted death and disability-adjusted life years (DALYs) rates, the prevalence of IHD increased. This trend can be attributed to various factors, including the aging population, increasing epidemic of risk factors such as diabetes, obesity, and low physical activity, as well as socio-economic disparities, poverty, limited screening and early detection of risk factors, war, and weak health infrastructure [1,2].

The age-standardized prevalence of IHD in the EMR was higher than the global level, particularly in high and middle socio-demographic index (SDI) countries. This aligns with other studies that have reported a higher prevalence of IHD in males compared to females [3,4]. The increasing prevalence of IHD in the EMR may be influenced by factors such as a lack of educational and community-based intervention programs for risk factor screening, management, and control, as well as the high prevalence of risk factors mentioned above [5]. The rapid socioeconomic transition and unhealthy lifestyle observed in the Gulf countries of the EMR have contributed to the burden of IHD [6]. Furthermore, the EMR has shown higher rates of risk factors such as high blood pressure, high cholesterol, physical inactivity, and obesity compared to other regions [7–13] ff. These numbers highlight the need for policymakers to prioritize preventive strategies and promote healthy lifestyles in the EMR countries. Better control of IHD risk factors through community education and screening programs can help reduce the prevalence of IHD.

The age-standardized death rate for IHD in the EMR was 1.78 times higher than the global level. High-income countries reported most considerable improvement in IHD death rates [22,23]. While high-income EMR countries reported the lowest age-standardized death rates in 2019, low-income countries in the region experienced higher rates. Improvements in healthcare systems, including increased access to evidence-based medications and cardiac interventions, have contributed to lower death rates in high-income countries [14–17]. However, challenges such as deficiencies in healthcare workforce, disparities between private and public healthcare practices, and limited access to essential medications still persist in many EMR countries [11,15,16,18–20]. Disparities in the availability and utilization of advanced treatments and interventions for IHD contribute to the variation in death rates among EMR countries. Countries with high mortality rates, such as Pakistan, need to reassess their strategies for IHD treatment.

The age-standardized DALYs of IHD in the EMR were almost twice as high as the global level, indicating the need for increased attention to preventive and treatment strategies. The slower decline in age-standardized DALY rates in the EMR compared to the global trend can be attributed to delayed policies and unorganized efforts in most countries [8]. Similar to other studies, males in the EMR had higher age-standardized DALY rates than females, as men generally develop IHD at a younger age and have a higher risk of coronary heart disease [21]. Bahrain exhibited the most significant improvement in all burden indicators, highlighting its success as a role model for other EMR countries. Policymakers in the region need to focus on well-designed preventive programs, well-equipped hospitals, and secondary prevention and rehabilitation programs, as well as develop comprehensive national policies and action plans for better control of IHD prevalence, death, and risk factors [21,24]. On the other hand, Pakistan showed an unfavorable trend in death rates and DALYs, indicating the need for a comprehensive evaluation of their IHD treatment strategies.

The burden of IHD in lower-middle-income countries in the EMR remains high, primarily due to key risk factors such as high blood pressure, smoking, unhealthy dietary habits, obesity, and low physical activity [25–30]. Environmental factors such as particulate matter pollution also contribute to the burden of IHD in the region. The distribution of risk factors and their effects on the burden of IHD vary among EMR countries. One of relative successful preventive programs in the region is tobacco control i.e. the prevalence of tobacco is lower than other WHO regions but it has increased recently [31]. While high-middle SDI countries have invested resources in reducing these risk factors, lower SDI countries face challenges due to limited resources and lower survival rates from other diseases [9,11]. These countries require more attention to preventive and therapeutic strategies and the development of suitable infrastructure in primary healthcare systems for better control of IHD risk factors.

Comprehensive education, risk factor control, and secondary prevention with evidence-based treatment are crucial for reducing the burden of IHD and recurrent events. However, suboptimal management of the disease after hospital discharge has been observed in the EMR [32,33]. The availability of cardiac rehabilitation (CR) programs for IHD patients in the EMR is limited, despite the proven cost-benefit of CR in reducing the burden of IHD [34–37]. The region requires more implementation of CR programs to improve outcomes for IHD patients.

While most EMR countries have access to treatment modalities such as angiography and angioplasty, the focus on prevention and rehabilitation in community health centers is inadequate. Therefore, there is a pressing need for the implementation of evidence-based medicine and cost-effective primary and secondary interventions to mitigate the impact of IHD in the EMR. Collaboration between governments, private sectors, and social media platforms can play a significant role in promoting healthy lifestyles and reducing IHD risk factors prevalent in the EMR countries. Community-based educational programs, the private sector, and social media involvement are essential in raising awareness and promoting behavioral changes.

Additionally, policymakers in the EMR should prioritize the establishment of well-qualified and registered data systems for IHD and risk factor surveillance. Accurate and reliable data collection in the healthcare system is crucial for informed decision-making and effective management of IHD.

In summary, addressing the burden of IHD in the EMR requires a comprehensive approach that combines prevention, treatment, and data-driven strategies. By implementing targeted interventions and improving access to quality healthcare services, the region can make significant progress in reducing the impact of IHD and improving the overall cardiovascular health of its population.

The findings of this study have important policy implications for addressing the growing burden of non-communicable diseases, particularly IHD, in the Eastern Mediterranean Region (EMR). By establishing the trend of IHD burden and comparing it with other countries and regions, policymakers can gain valuable insights for resource allocation and priority setting in IHD prevention, control, and treatment. Previous research has highlighted that EMR countries with higher age-standardized DALYs for IHD tend to have lower healthcare access and quality index scores, indicating the need for improved healthcare facilities [38]. Furthermore, it has been observed that medical advancements in high-income countries have increased life expectancy, resulting in a higher prevalence of IHD but lower mortality rates. On the other hand, individuals in middle socioeconomic ranges may face barriers to accessing optimal treatment modalities, leading to higher mortality rates compared to high-income populations [39].

In the EMR, a significant portion of the population lives below the international poverty line, and there are variations in literacy rates and access to healthcare services. Many low-income countries struggle to provide quality healthcare services to their entire population, leading to delays in diagnosis, inadequate prevention efforts, and poorer health outcomes. Furthermore, individuals in low-income brackets may experience higher mortality rates from other diseases before reaching old age, further impacting the burden of IHD in the region.

Therefore, policy interventions should focus on addressing the socioeconomic disparities and improving access to quality healthcare services in the EMR. Efforts should be directed towards implementing effective prevention strategies, ensuring timely diagnosis, and providing optimal treatment modalities for individuals at risk of or affected by IHD. Additionally, targeted interventions should be developed to address the specific challenges faced by low-income countries in the region, aiming to alleviate economic burdens and improve healthcare outcomes for their populations.

### Limitations

This study has several limitations that should be acknowledged. Firstly, the absence of data for Palestine limited the inclusion of this country in our analyses, which may have impacted the overall findings. Additionally, while we assessed the availability of cardiac rehabilitation (CR) services, we did not evaluate the quality of these programs. Future studies should consider assessing the quality and effectiveness of CR services in the Eastern Mediterranean Region (EMR). Another limitation is the lack of a comprehensive analysis of the lag time between disability-adjusted life years (DALYs) and the healthcare access and quality index (HAQ). In this study, we examined the relationship between HAQ and DALYs in 2019 for a general overview, but further investigations should explore the dynamic interplay between these factors over time. The other restriction is lack of analysis of world excluding EMR countries with EMR countries. Lastly, the study did not conduct a decomposition analysis to determine the specific contributions of changing demographics, population growth, aging, and risk factors to the burden of ischemic heart disease (IHD). Our result is descriptive without forecasting.

## Conclusion

In conclusion, our study reveals an increase in age-standardized prevalence of ischemic heart disease (IHD) and a decrease in age-standardized DALYs and death rates in the Eastern Mediterranean Region (EMR). However, despite these improvements, the burden of IHD in the EMR remains higher than the global levels. It underscores the need for comprehensive preventive and treatment strategies, including the control of risk factors, implementation of evidence-based interventions, improvement of healthcare systems, and the availability of CR programs. There is a clear need for interventions and strategies aimed at reducing the incidence and prevalence of IHD, as well as improving treatment modalities to reduce mortality and overall burden of the disease in this region.

## Supporting information

S1 File Trend of all age deaths, disability-adjusted life years (DALYs), and prevalence of IHD among males and females during 1990 to 2019 worldwide and in EMR. (DOCX)

S2 File Age-standardized of prevalence percentage (a.), death rate (b.) DALYs rate(c.) of IHD patients (per 100,000) based on different age groups in 1990 and 2019.
(DOCX)

S3 File Comparison of age-standardized prevalence percentage of IHD (per 100,000) for males in 1990,2005 and 2019, and their relative percentage change by SDI status and EMR countries. (DOCX)

S4 File Table. Comparison of age-standardized prevalence percentage of IHD (per 100,000) for females in 1990,2005 and 2019, and their relative percentage change by SDI status and EMR countries. (DOCX)

S5 File Table. Comparison of age-standardized death rate of IHD (per 100,000) for males in 1990,2005 and 2019, and their relative percentage change by SDI level and EMR countries. (DOCX)

S6 File Comparison of age-standardized death rate of IHD (per 100,000) for females in 1990,2005 and 2019, and their relative percentage change by SDI level and EMR countries. (DOCX)

S7 File Comparison of age-standardized disability-adjusted life years (DALY) rate of IHD (per 100,000) for males in 1990,2005 and 2019, and their relative percentage change by SDI level and EMR countries. (DOCX)

S8 File Comparison of age-standardized disability-adjusted life years (DALYs) rate of IHD (per 100,000) for females in 1990,2005 and 2019, and their relative percentage change by SDI status and EMR countries. (DOCX)

S9 File Comparison of age-standardized DALY attribute risk factors (metabolic, behivioral and enviormental) rate of IHD (per 100,000) in 1990,2005 and 2019 by SDI level and EMR countries. (DOCX)

S10 File Comparison of three DALYs attributed behavioral risk factors tobacco, dietary risk and low physical activity by SDI level and geographical area. (DOCX)

S11 File Comparison of disability-adjusted life years (DALYs) attribute risk factors in global and EMR countries in 3-point time 1990,2005 and 2019. (DOCX)

S12 File The relationship healthcare access and quality index (HAQ) and DALY by EMR countries. (DOCX)
