# Supplementary material for: The burden of ischemic heart disease and the epidemiologic transition in the Eastern Mediterranean Region: 1990–2019
Source: PLoS One. 2023 Sep 5;18(9):e0290286. doi: 10.1371/journal.pone.0290286 (PMC10479892; doi:10.1371/journal.pone.0290286)
Supplement: S1 File — (DOCX) [file pone.0290286.s001.docx]

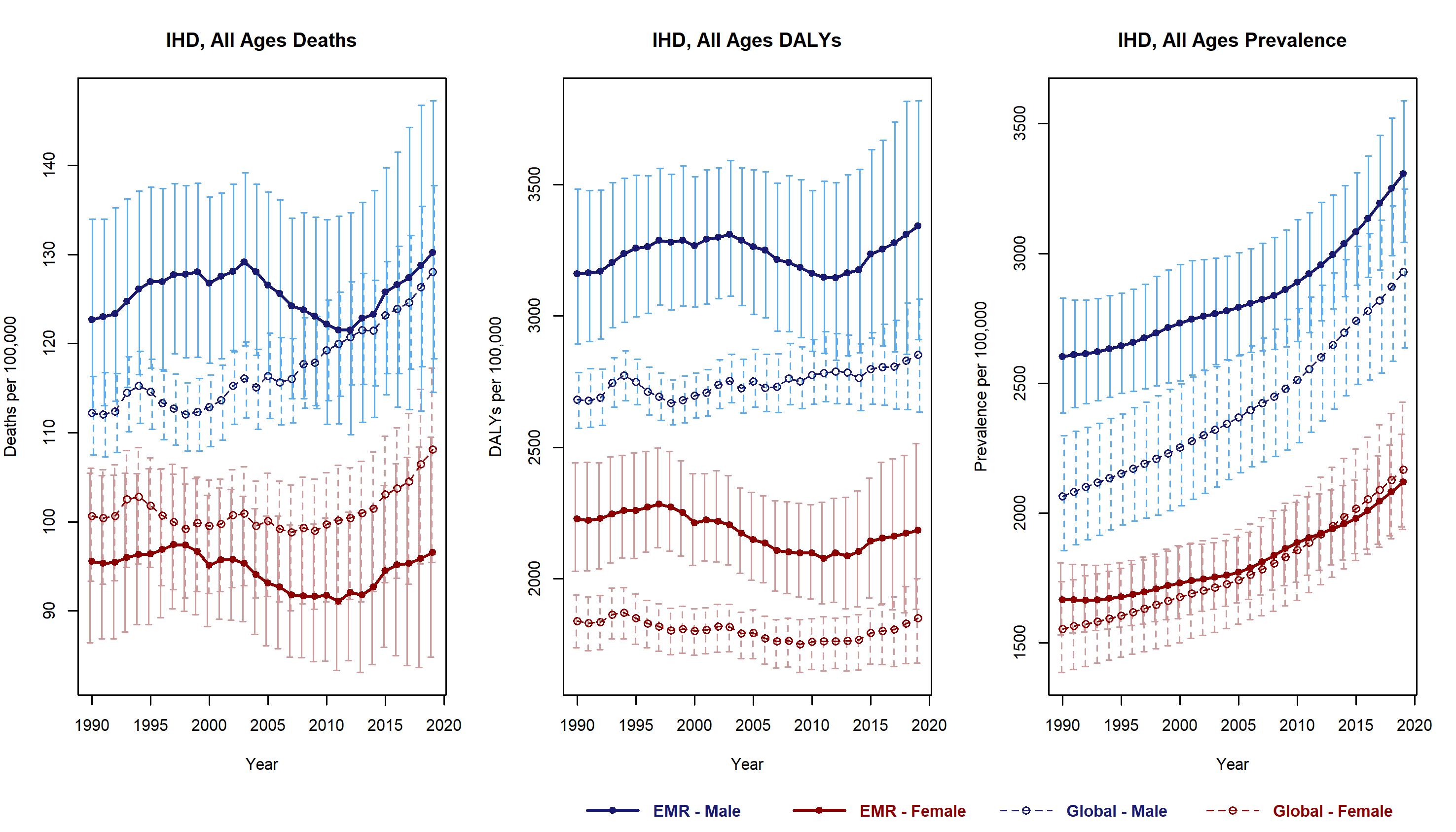


S1. Trend of all age deaths, disability-adjusted life years (DALYs), and prevalence of IHD among males and females during 1990 to 2019 worldwide and in EMR
