# Supplementary material for: The burden of ischemic heart disease and the epidemiologic transition in the Eastern Mediterranean Region: 1990–2019
Source: PLoS One. 2023 Sep 5;18(9):e0290286. doi: 10.1371/journal.pone.0290286 (PMC10479892; doi:10.1371/journal.pone.0290286)
Supplement: S2 File — Age-standardized of prevalence percentage (a.), death rate (b.) DALYs rate(c.) of IHD patients (per 100,000) based on different age groups in 1990 and 2019. (DOCX) [file pone.0290286.s002.docx]

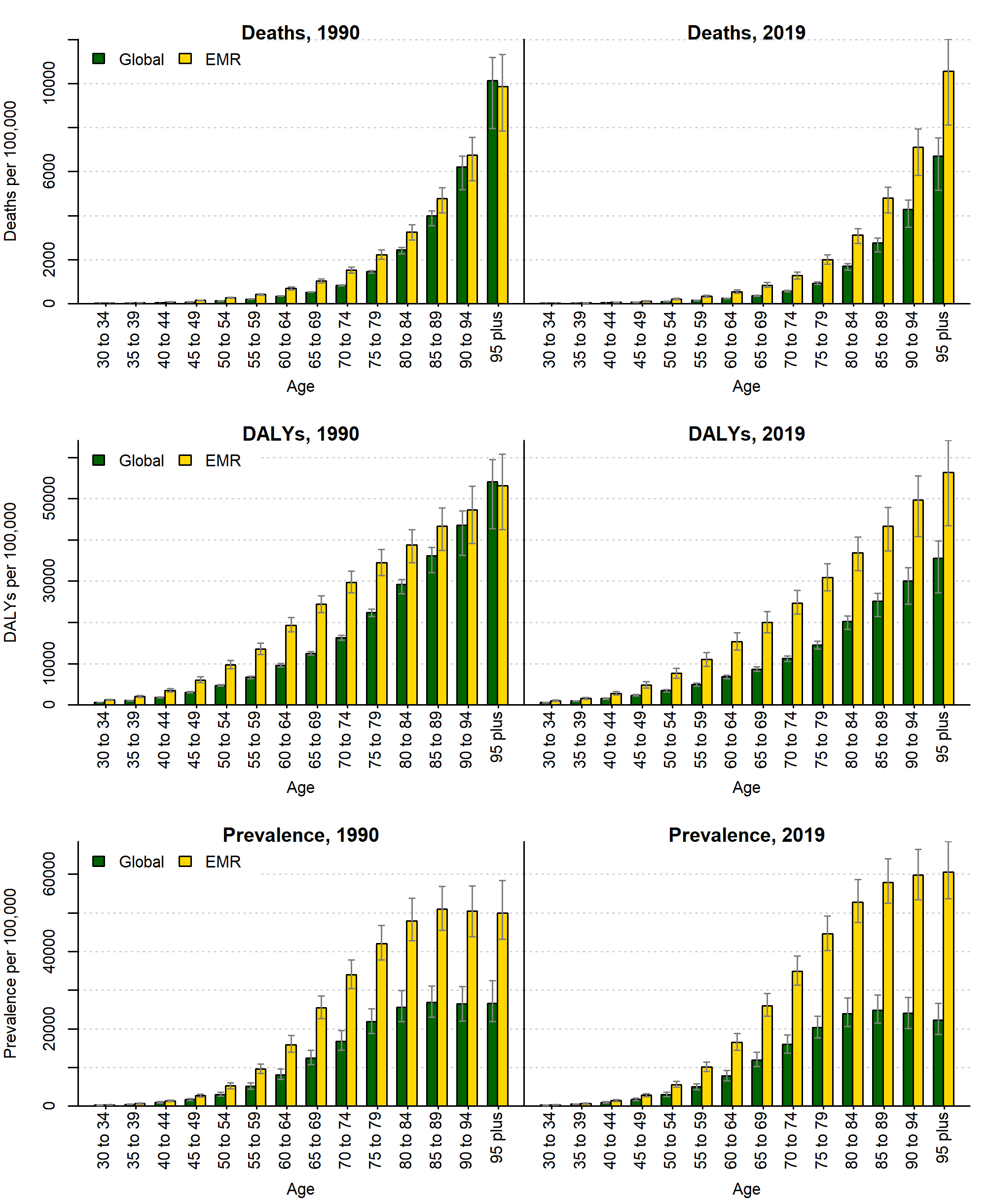


S2. Age-standardized of prevalence percentage (a.), death rate (b.) DALYs rate(c.) of IHD patients (per 100,000) based on different age groups in 1990 and 2019.
