# Supplementary material for: The burden of ischemic heart disease and the epidemiologic transition in the Eastern Mediterranean Region: 1990–2019
Source: PLoS One. 2023 Sep 5;18(9):e0290286. doi: 10.1371/journal.pone.0290286 (PMC10479892; doi:10.1371/journal.pone.0290286)
Supplement: S3 File — (DOCX) [file pone.0290286.s003.docx]

S3. Comparison of age-standardized prevalence percentage of IHD (per 100,000) for **males in** 1990,2005 and 2019, and their relative percentage change by SDI status and EMR countries.

| SDI | Countries | Prevalence percentage(95%UI) | | | %Δ ($\frac{x_{i+1}-x_{i}}{x_{i}})$ | | |
| --- | --- | --- | --- | --- | --- | --- | --- |
|  |  | 1990 | 2005 | 2019 | 1990-2005 | 2005-2019 | 1990-2019 |
| - | Global | 3.36(3.02-3.72) | 3.24(2.97-3.57) | 3.18 (2.87-3.52) | -3.57 | -1.85 | -5.36 |
|  | EMR | 5.89(5.41-6.39) | 6.27(5.86-6.74) | 6.44(5.93-6.97) | 6.45 | 2.71 | 9.34 |
| High | Kuwait | 6.64 (6.12-7.18) | 6.94(6.50-7.46) | 6.97(6.43-7.57) | 4.52 | 0.43 | 4.97 |
|  | United Arab Emirates | 5.84 (5.39-6.33) | 5.9(5.52-6.28) | 6.16(5.69-6.68) | 1.03 | 4.41 | 5.48 |
|  | Qatar | 5.83 (5.38-6.3) | 5.85(5.46-6.29) | 5.72(5.27-6.2) | 0.34 | -2.22 | -1.89 |
| High middle | Libya | 5.65 (5.23-6.12) | 6.09(5.68-6.53) | 6.25(5.77-6.78) | 7.79 | 2.63 | 10.62 |
|  | Jordan | 6.67 (6.13-7.26) | 6.64(6.17-7.17) | 6.56(6.04-7.15) | -0.45 | -1.20 | -1.65 |
|  | Saudi Arabia | 5.6 (5.17-6.05) | 6.28(5.87-6.73) | 6.40(5.93-6.91) | 12.14 | 1.91 | 14.29 |
|  | Lebanon | 6.21(5.75-6.70) | 6.14(5.73-6.57) | 6.60(6.09-7.12) | -1.13 | 7.49 | 6.28 |
|  | Bahrain | 6.72 (6.19-7.30) | 6.32(5.91-6.78) | 6.37 (5.89-6.93) | -5.95 | 0.79 | -5.21 |
|  | Oman | 6.03 (5.56-6.57) | 6.55(6.11-7.03) | 6.86(6.34-7.41) | 8.62 | 4.73 | 13.76 |
| Middle | Tunisia | 5.52 (5.08-5.99) | 5.71(5.33-6.12) | 5.81(5.36-6.29) | 3.44 | 1.75 | 5.25 |
|  | Iran (Islamic Republic of) | 7.89 (7.2-8.64) | 8.06(7.43-8.72) | 8.07(7.36-8.83) | 2.15 | 0.12 | 2.28 |
|  | Iraq | 7.04 (6.5-7.64) | 6.88(6.44-7.41) | 6.9(6.36-7.48) | -2.27 | 0.29 | -1.99 |
|  | Syrian Arab Republic | 6.11 (5.66-6.59) | 6.38(5.97-6.79) | 6.55(6.08-7.05) | 4.42 | 2.66 | 7.20 |
|  | Egypt | 6.60 (6.16-7.11) | 6.56(6.19-7.00) | 6.84(6.35-7.36) | -0.61 | 4.27 | 3.64 |
| Low middle | Djibouti | 2.76 (2.43-3.13) | 2.96(2.61-3.38) | 3.05(2.69-3.47) | 7.25 | 3.04 | 10.51 |
|  | Morocco | 6.71 (6.2-7.29) | 6.83(6.35-7.39) | 6.70(6.19-7.29) | 1.79 | -1.90 | -0.15 |
|  | Sudan | 6.03 (5.59-6.51) | 6.12(5.73-6.58) | 6.29(5.8-6.77) | 1.49 | 2.78 | 4.31 |
| Low | Somalia | 2.42 (2.13-2.77) | 2.54(2.24-2.91) | 2.59(2.27-2.93) | 4.96 | 1.97 | 7.02 |
|  | Pakistan | 4.57 (4.09-5.09) | 5.13(4.64-5.67) | 5.29(4.74-5.89) | 12.25 | 3.12 | 15.75 |
|  | Yemen | 5.91 (5.48-6.38) | 5.89(5.52-6.31) | 6.07(5.62-6.56) | -0.34 | 3.06 | 2.71 |
|  | Afghanistan | 6.46(5.99-6.97) | 6.41(5.98-6.91) | 6.55(6.05-7.12) | -0.77 | 2.18 | 1.39 |

**^*^**95% uncertainty intervals (UI) gathered from GBD website.
