# Supplementary material for: The burden of ischemic heart disease and the epidemiologic transition in the Eastern Mediterranean Region: 1990–2019
Source: PLoS One. 2023 Sep 5;18(9):e0290286. doi: 10.1371/journal.pone.0290286 (PMC10479892; doi:10.1371/journal.pone.0290286)
Supplement: S4 File — (DOCX) [file pone.0290286.s004.docx]

S4. Comparison of age-standardized prevalence percentage of IHD (per 100,000) for fe**males in** 1990,2005 and 2019, and their relative percentage change by SDI status and EMR countries

| SDI | Countries | Prevalence percentage(95%UI) | | | %Δ ($\frac{x_{i+1}-x_{i}}{x_{i}})$ | | |
| --- | --- | --- | --- | --- | --- | --- | --- |
|  |  | 1990 | 2005 | 2019 | 1990-2005 | 2005-2019 | 1990-2019 |
| - | Global | 2.03(1.81-2.27) | 1.96(1.77-2.18) | 1.97(1.76-2.21) | -3.45 | 0.51 | -2.96 |
|  | EMR | 3.94(3.63-4.28) | 4.08(3.80-4.37) | 4.13(3.81-4.48) | 3.55 | 1.23 | 4.82 |
| High | Kuwait | 4.45(4.11-4.81) | 4.63(4.33-4.94) | 4.59(4.26-4.94) | 4.04 | -0.86 | 3.15 |
|  | United Arab Emirates | 3.85 (3.56-4.15) | 3.87(3.63-4.11) | 4.01(3.69-4.33) | 0.52 | 3.62 | 4.16 |
|  | Qatar | 3.79(3.49-4.11) | 3.79(3.55-4.06) | 3.66(3.38-3.95) | 0.00 | -3.43 | -3.43 |
| High middle | Libya | 3.73 (3.44-4.02) | 3.98(3.72-4.26) | 4.05(3.74-4.38) | 6.70 | 1.76 | 8.58 |
|  | Jordan | 4.35 (4.01-4.70) | 4.30(4.02-4.61) | 4.21(3.88-4.55) | -1.15 | -2.09 | -3.22 |
|  | Saudi Arabia | 3.76 (3.49-4.06) | 4.14(3.87-4.41) | 4.16(3.85-4.49) | 10.11 | 0.48 | 10.64 |
|  | Lebanon | 4.01 (3.70-4.32) | 3.97(3.73-4.25) | 4.17(3.88-4.51) | -1.00 | 5.04 | 3.99 |
|  | Bahrain | 4.46(4.12-4.82) | 4.19(3.92-4.47) | 4.14(3.82-4.49) | -6.05 | -1.19 | -7.17 |
|  | Oman | 4.05 (3.74-4.38) | 4.33(4.05-4.62) | 4.52(4.18-4.86) | 6.91 | 4.39 | 11.60 |
| Middle | Tunisia | 3.63(3.53-3.93) | 3.73(3.50-3.98) | 3.76(3.48-4.06) | 2.75 | 0.80 | 3.58 |
|  | Iran (Islamic Republic of) | 5.16 (4.66-5.69) | 5.19(4.77-5.66) | 4.98(4.5-5.15) | 0.58 | -4.05 | -3.49 |
|  | Iraq | 4.7 (4.35-5.08) | 4.50(4.22-4.81) | 4.51(4.18-4.89) | -4.26 | 0.22 | -4.04 |
|  | Syrian Arab Republic | 3.98 (3.69-4.29) | 4.14(3.89-4.41) | 4.23(3.95-4.57) | 4.02 | 2.17 | 6.28 |
|  | Egypt | 4.57(4.26-4.91) | 4.58(4.32-4.86) | 4.8(4.49-5.14) | 0.22 | 4.80 | 5.03 |
| Low middle | Djibouti | 1.52 (1.37-1.70) | 1.61(1.47-1.79) | 1.64(1.47-1.82) | 5.92 | 1.86 | 7.89 |
|  | Morocco | 4.37(4.02-4.72) | 4.44(4.15-4.73) | 4.3(3.98-4.66) | 1.60 | -3.15 | -1.60 |
|  | Sudan | 3.97(3.66-4.25) | 4.04(3.78-4.31) | 4.16(3.84-4.48) | 1.76 | 2.97 | 4.79 |
| Low | Somalia | 1.36 (1.22-1.51) | 1.41(1.27-1.57) | 1.43(1.28-1.59) | 3.68 | 1.42 | 5.15 |
|  | Pakistan | 2.95(2.62-3.3) | 3.10(2.80-3.47) | 3.22(2.88-3.61) | 5.08 | 3.87 | 9.15 |
|  | Yemen | 3.89(3.6-4.19) | 3.89(3.64-4.16) | 3.99(3.69-4.31) | 0.00 | 2.57 | 2.57 |
|  | Afghanistan | 4.36 (4.03-4.69) | 4.33(4.05-4.63) | 4.39(4.04-4.77) | -0.69 | 1.39 | 0.69 |

**^*^**95% uncertainty intervals (UI) gathered from GBD website.
