# Supplementary material for: The burden of ischemic heart disease and the epidemiologic transition in the Eastern Mediterranean Region: 1990–2019
Source: PLoS One. 2023 Sep 5;18(9):e0290286. doi: 10.1371/journal.pone.0290286 (PMC10479892; doi:10.1371/journal.pone.0290286)
Supplement: S5 File — (DOCX) [file pone.0290286.s005.docx]

S5. Comparison of age-standardized death rate of IHD (per 100,000) for **males in** 1990,2005 and 2019, and their relative percentage change by SDI level and EMR countries.

| SDI | Countries | Death Rate (95%UI) | | | %Δ ($\frac{x_{i+1}-x_{i}}{x_{i}})$ | | |
| --- | --- | --- | --- | --- | --- | --- | --- |
|  |  | 1990 | 2005 | 2019 | 1990-2005 | 2005-2019 | 1990-2019 |
| - | Global | 205.24(194.29-213.05) | 141.23(131.93-147.32) | 144.6(132.87-154.96) | -31.19 | 2.39 | -29.55 |
|  | EMR | 286.76(261.82-312.99) | 263.91(244.39-282.68) | 260.51(230.02-291.48) | -7.97 | -1.29 | -9.15 |
| High | Kuwait | 202.69(182.12-219.44) | 146.41(133.73-153.86) | 142.92(116.01-174.1) | -27.77 | -2.38 | -29.49 |
|  | United Arab Emirates | 308.48(249.54-388.62) | 326.04(276.62-387.52) | 187.24(140.05-240.61) | 5.69 | -42.57 | -39.30 |
|  | Qatar | 458.5(357.61-547.54) | 327.93(286.74-368.86) | 230.47(181.69-283.23) | -28.48 | -29.72 | -49.73 |
| High middle | Libya | 204.65(158.62-271.43) | 154.84(128.78-201.14) | 188.13(142.89-262.59) | -24.34 | 21.50 | -8.07 |
|  | Jordan | 226.57(193.21-264.01) | 182.76(162.68-201.34) | 135.08(108.11-167.19) | -19.34 | -26.09 | -40.38 |
|  | Saudi Arabia | 255.34(202.38-306.49) | 259.05(237.54-281.32) | 225.63(191.21-261.37) | 1.45 | -12.90 | -11.64 |
|  | Lebanon | 432.53(367.55-507.12) | 264.43(210.2-296.66) | 322.56(238.37-370.2) | -38.86 | 21.98 | -25.42 |
|  | Bahrain | 488.78(434.14-544.59) | 280.79(253.64-310.28) | 159.29(128.99-196.33) | -42.55 | -43.27 | -67.41 |
|  | Oman | 524.28(422.98-634.68) | 410.40(380.67-434.34) | 361.87(317.2-414.69) | -21.72 | -11.83 | -30.98 |
| Middle | Tunisia | 281.79(242.57-328.65) | 225.69(180.71-278.33) | 237.39(179.63-302.5) | -19.91 | 5.18 | -15.76 |
|  | Iran (Islamic Republic of) | 322.07(295.45-352.94) | 223.38(205.52-235.21) | 177.18(161.53-192.51) | -30.64 | -20.68 | -44.99 |
|  | Iraq | 356.78(293.2-422.99) | 282.37(228.59-345.26) | 304.61(249.2-349.64) | -20.86 | 7.88 | -14.62 |
|  | Syrian Arab Republic | 414.67(339.49-496.65) | 361.5(322.04-399.96) | 376.36(295.18-477.86) | -12.82 | 4.11 | -9.24 |
|  | Egypt | 425.56(382.87-470.54) | 391(356.68-425.62) | 349.62(269.48-445 | -8.12 | -10.58 | -17.84 |
| Low middle | Djibouti | 119.8(90.87-155.08) | 128.58(92.11-174.25) | 129.15(89.66-177.31) | 7.33 | 0.44 | 7.80 |
|  | Morocco | 345.77(291.42-407.81) | 268.38(223.51-321.12) | 314.01(247.68-366.75) | -22.38 | 17.00 | -9.19 |
|  | Sudan | 412.63(318.49-506.18) | 300.27(224.83-377.85) | 298.45(220.32-385.84) | -27.23 | -0.61 | -27.67 |
| Low | Somalia | 152.05(113.16-191.72) | 119.84(90.71-155.18) | 151.13(110.84-197.4) | -21.18 | 26.11 | -0.61 |
|  | Pakistan | 158.31(128.84-192.36) | 199.52(175.96-225.5) | 226.53(172.92-285.14) | 26.03 | 13.54 | 43.09 |
|  | Yemen | 420.89(330.75-529.68) | 300.18(240.83-369.32) | 336.79(265.1-433.95) | -28.68 | 12.20 | -19.98 |
|  | Afghanistan | 439.29(351.44-537.61) | 382.71(307.8-472.38) | 326.72(254.48-390.43) | -12.88 | -14.63 | -25.63 |

**^*^**95% uncertainty intervals (UI) gathered from GBD website.
