# Supplementary material for: The burden of ischemic heart disease and the epidemiologic transition in the Eastern Mediterranean Region: 1990–2019
Source: PLoS One. 2023 Sep 5;18(9):e0290286. doi: 10.1371/journal.pone.0290286 (PMC10479892; doi:10.1371/journal.pone.0290286)
Supplement: S6 File — (DOCX) [file pone.0290286.s006.docx]

S6. Comparison of age-standardized death rate of IHD (per 100,000) for fe**males in** 1990,2005 and 2019, and their relative percentage change by SDI level and EMR countries

| SDI | Countries | Death Rate (95%UI) | | | %Δ ($\frac{x_{i+1}-x_{i}}{x_{i}})$ | | |
| --- | --- | --- | --- | --- | --- | --- | --- |
|  |  | 1990 | 2005 | 2019 | 1990-2005 | 2005-2019 | 1990-2019 |
| - | Global | 141.73(130-149.72) | 115.44(104.95-121.93) | 95.07(83.91-103.11) | -18.55 | -17.65 | -32.92 |
|  | EMR | 242.8(218.2-268.45) | 234.05(214.48-253.82) | 209.06(184.9-235.87) | -3.60 | -10.68 | -13.90 |
| High | Kuwait | 173.91(154.21-192.53) | 89.94(78.97-97.11) | 58.58(46.35-71.02) | -48.28 | -34.87 | -66.32 |
|  | United Arab Emirates | 270.69 (214.76-342.94) | 368.82(317.84-437.81) | 143.14(113.9-182.12) | 36.25 | -61.19 | -47.12 |
|  | Qatar | 348.47 (260.76-418.46) | 381.72(329.52-434.00) | 332.93(281.55-387.27) | 9.54 | -12.78 | -4.46 |
| High middle | Libya | 173.28 (139.7-209.39) | 133.31(109.33-159.27) | 153.84(120.45-195.53) | -23.07 | 15.40 | -11.22 |
|  | Jordan | 222.32 (190.25-256.56) | 210.73(191.45-290.92) | 107.79(87.86-129.37) | -5.21 | -48.85 | -51.52 |
|  | Saudi Arabia | 221.97 (181.06-266.68) | 227.99(205.18-245.72) | 174.91(142.02-213.10) | 2.71 | -23.28 | -21.20 |
|  | Lebanon | 276.6 (234.78-323.64) | 210.73(186.62-236.63) | 174.97(113.83-202.57) | -23.81 | -16.97 | -36.74 |
|  | Bahrain | 388.09 (342.49-430.03) | 258.5(229.23-288.09) | 147.31(122.31-174.42) | -33.39 | -43.01 | -62.04 |
|  | Oman | 417.21 (339.43-501.2) | 367.32(336.52-392.84) | 299.99(264.68-338.67) | -11.96 | -18.33 | -28.10 |
| Middle | Tunisia | 197.18 (163.61-228.43) | 177.56(139.04-221.18) | 154.27(114-194.23) | -9.95 | -13.12 | -21.76 |
|  | Iran (Islamic Republic of) | 245.55 (213.62-269.07) | 193.52(174.91-204.22) | 150.93(133.83-164) | -21.19 | -22.01 | -38.53 |
|  | Iraq | 267.16 (223.53-313.13) | 237.37(191.45-290.92) | 209.49(176.41-245.4) | -11.15 | -11.75 | -21.59 |
|  | Syrian Arab Republic | 359.14(297.07-419.32) | 381.55(331.96-427.54) | 369.6(305.32-446.55) | 6.24 | -3.13 | 2.91 |
|  | Egypt | 395.62 (355.76-448.82) | 405.54(359-450.62) | 404.9(314.2-486.81) | 2.51 | -0.16 | 2.35 |
| Low middle | Djibouti | 80.66 (63.54-101.1) | 92.64(68.57-124.48) | 100.15(70.67-137.28) | 14.85 | 8.11 | 24.16 |
|  | Morocco | 270.17 (231.27-310.75) | 264.65(220.56-317.05) | 244.16(199.36-287.72) | -2.04 | -7.74 | -9.63 |
|  | Sudan | 307.85(249.57-371.99) | 255.92(193.88-327.80) | 240.43(190.58-301.85) | -16.87 | -6.05 | -21.90 |
| Low | Somalia | 85.21(56.19-120.22) | 96.58(64.96-137.80) | 104.52(73.02-148.1) | 13.34 | 8.22 | 22.66 |
|  | Pakistan | 131.29(101.2-163.11) | 157.54(132.88-187.06) | 150.52(119.88-190.77) | 19.99 | -4.46 | 14.65 |
|  | Yemen | 296.82(242.82-360.2) | 255.74(209.99-309.13) | 254.67(208.47-321.02) | -13.84 | -0.42 | -14.20 |
|  | Afghanistan | 379.11(310.56-458.721) | 360.74(279.53-453.33) | 316.39(246.6-396.67) | -4.85 | -12.29 | -16.54 |

**^*^**95% uncertainty intervals (UI) gathered from GBD website.
