# Supplementary material for: The burden of ischemic heart disease and the epidemiologic transition in the Eastern Mediterranean Region: 1990–2019
Source: PLoS One. 2023 Sep 5;18(9):e0290286. doi: 10.1371/journal.pone.0290286 (PMC10479892; doi:10.1371/journal.pone.0290286)
Supplement: S7 File — (DOCX) [file pone.0290286.s007.docx]

S7. Comparison of age-standardized disability-adjusted life years (DALY) rate of IHD (per 100,000) for **males in** 1990,2005 and 2019, and their relative percentage change by SDI level and EMR countries

| SDI | Countries | DALYs Rate (95%UI) | | | %Δ ($\frac{x_{i+1}-x_{i}}{x_{i}})$ | | |
| --- | --- | --- | --- | --- | --- | --- | --- |
|  |  | 1990 | 2005 | 2019 | 1990-2005 | 2005-2019 | 1990-2019 |
| - | Global | 4003.52(3840.47-4147.26) | 2681.06(2571.04-2788.57) | 2899.51(2681.07-3117.87) | -33.03 | 8.15 | -27.58 |
|  | EMR | 6181.11(5681.89-6771.8) | 6168.4(5694.57-6676.43) | 5386.78(4794.97-6106.79) | -0.21 | -12.67 | -12.85 |
| High | Kuwait | 4449.16(4099.9-4815.85) | 4003.86(3811.34-4166.32) | 3155.4(2559.13-3861) | -10.01 | -21.19 | -29.08 |
|  | United Arab Emirates | 6035.91(4744.6-7752.52) | 5468.53(4486.71-6723.98) | 3719.1(2743.02-4896.77) | -9.40 | -31.99 | -38.38 |
|  | Qatar | 8025.65(6422.56-9729.13) | 5070.88(4138.28-6095.29) | 3336.97(2583.5-4159.53) | -36.82 | -34.19 | -58.42 |
| High middle | Libya | 4221.9(3340.06-5633.77) | 3695.36(3153.59-5307.48) | 3875.95(2956.86-5525.91) | -12.47 | 4.89 | -8.19 |
|  | Jordan | 4875.82(4162.53-5710.61) | 3535.22(3057.6-4058.75) | 2764.59(2211.87-3442.75) | -27.49 | -21.80 | -43.30 |
|  | Saudi Arabia | 5196.92(4026.64-6416.05) | 5969.27(5464.53-6602.01) | 4773.66(3932.71-5703.8) | 14.86 | -20.03 | -8.14 |
|  | Lebanon | 9031.96(7616.27-10657.7) | 6299.32(5184.76-7256.58) | 6370.29(4826.03-7395.43) | -30.26 | 1.13 | -29.47 |
|  | Bahrain | 9408.42(8253.2-10609.41) | 5261.65(4658.46-5924.37) | 2742.79(2208.47-3370.99) | -44.08 | -47.87 | -70.85 |
|  | Oman | 10298.85(8083.35-12722.04) | 9077.65(8440.97-9729.39) | 5903.44(5139.67-6794.82) | -11.86 | -34.97 | -42.68 |
| Middle | Tunisia | 5277.85(4499.96-6190.81) | 5029.35(3787.05-6402.35) | 4305.26(3177.36-5622.31) | -4.71 | -14.40 | -18.43 |
|  | Iran (Islamic Republic of) | 6502.03(6020.94-7142.42) | 4923.86(4686.32-5352.12) | 3393.61(3160.62-3734.03) | -24.27 | -31.08 | -47.81 |
|  | Iraq | 7538.87(6167.14-9159.61) | 6966.88(5401.69-8804.28) | 5941.16(4737.96-7044.15) | -7.59 | -14.72 | -21.19 |
|  | Syrian Arab Republic | 9096.19(7352.81-11076.1) | 7582.21(6441.19-8937.87) | 7468.63(5765.19-9771.25) | -16.64 | -1.50 | -17.89 |
|  | Egypt | 8920.82(8076.99-10172.53) | 8088.70(7285.38-9290.76) | 7430.93(5596.04-9725.48) | -9.33 | -8.13 | -16.70 |
| Low middle | Djibouti | 2401.56(1783.32-3160.33) | 2658.31(1866-3632.10) | 2594.55(1769.83-3623.09) | 10.69 | -2.40 | 8.04 |
|  | Morocco | 7119.33(6017.75-8452.34) | 5768.77(4486.95-7280.09) | 5955.15(4578.27-7153.08) | -18.97 | 3.23 | -16.35 |
|  | Sudan | 8980.35(6855.16-11179.35) | 7181.11(5020.51-9495.20) | 6073.05(4375.87-8091.97) | -20.04 | -15.43 | -32.37 |
| Low | Somalia | 5196.92(4026.64-6416.05) | 3388.45(2542.1-4277.53) | 3347.37(2471.08-4389.85) | -34.80 | -1.21 | -35.59 |
|  | Pakistan | 3516.09(2895.27-4256.74) | 5498.44(4612.01-6546.47) | 5166.59(3843.78-6618.66) | 56.38 | -6.04 | 46.94 |
|  | Yemen | 9201.74(7060.29-11911.2) | 7327.36(5514.87-9574.75) | 6987.02(5365.16-9301.01) | -20.37 | -4.64 | -24.07 |
|  | Afghanistan | 10105.85(7917.48-12721.26) | 9353.60(7217.02-11625.67) | 7187.98(5568.05-8740.03) | -7.44 | -23.15 | -28.87 |

**^*^**95% uncertainty intervals (UI) gathered from GBD website.
