# Supplementary material for: The burden of ischemic heart disease and the epidemiologic transition in the Eastern Mediterranean Region: 1990–2019
Source: PLoS One. 2023 Sep 5;18(9):e0290286. doi: 10.1371/journal.pone.0290286 (PMC10479892; doi:10.1371/journal.pone.0290286)
Supplement: S8 File — (DOCX) [file pone.0290286.s008.docx]

S8. Comparison of age-standardized disability-adjusted life years ( DALYs) rate of IHD (per 100,000) for females in 1990,2005 and 2019, and their relative percentage change by SDI status and EMR countries

| SDI | Countries | DALYs Rate (95%UI) | | | %Δ ($\frac{x_{i+1}-x_{i}}{x_{i}})$ | | |
| --- | --- | --- | --- | --- | --- | --- | --- |
|  |  | 1990 | 2005 | 2019 | 1990-2005 | 2005-2019 | 1990-2019 |
| - | Global | 2366.31(2222.27-2491.26) | 2681.06(2571.04-2788.57) | 1637.86(1486.52-1769.83) | 13.30 | -38.91 | -30.78 |
|  | EMR | 4693(4274.61-5157.23) | 4392.57(4071.02-4752.32) | 3812.28(3331.03-4339.72) | -6.40 | -13.21 | -18.77 |
| High | Kuwait | 3163.34(2880.56-3499.07) | 1673.18(1534.82-1777.96) | 990.56(802.45-1202.78) | -47.11 | -40.80 | -68.69 |
|  | United Arab Emirates | 4366.26(3436.29-5521.96) | 5638.04(4828.82-6739.91) | 2322.33(1826.13-2985.71) | 29.13 | -58.81 | -46.81 |
|  | Qatar | 5900.44(4490.33-7128.41) | 6123.08(5272.61-7047.02) | 4426.66(3675.97-5241.37) | 3.77 | -27.71 | -24.98 |
| High middle | Libya | 3370.14(2802.49-4058.28) | 2569.02(2199.57-3008.09) | 2958.3(2325.96-3780.16) | -23.77 | 15.15 | -12.22 |
|  | Jordan | 3816.06(3245.39-4429.02) | 3373.35(2976.65-3798.75) | 1694.17(1389.93-2057.15) | -11.60 | -49.78 | -55.60 |
|  | Saudi Arabia | 4281.39(3470.88-5226.91) | 4541.421(4127.22-4933.17) | 3371.15(2731.57-4149.63) | 6.07 | -25.77 | -21.26 |
|  | Lebanon | 4849.26(4170.67-5684.16) | 3394.95(2603.19-3873.80) | 2923.97(1919.43-3445.72) | -29.99 | -13.87 | -39.70 |
|  | Bahrain | 6723.46(5942.54-7557.97) | 4131.80(3699.55-4619.66) | 2217.58(1846.35-2665.72) | -38.55 | -46.33 | -67.02 |
|  | Oman | 7651.27(6127.6-9425.75) | 6756.27(6265.43-7213.74) | 4848.08(4283.67-5477.32) | -11.70 | -28.24 | -36.64 |
| Middle | Tunisia | 3350.82(2815.39-3892.28) | 2908.77(2278.62-3670.07) | 2465.13(1822.86-3136.19) | -13.19 | -15.25 | -26.43 |
|  | Iran (Islamic Republic of) | 4260.75(3841.22-4634.4) | 3143.68(2928.11-3288.16) | 2298.41(2100.47-2483.96) | -26.22 | -26.89 | -46.06 |
|  | Iraq | 5042.75(4222.49-5983.1) | 4482.83(3546.75-5617.01) | 3644.75(3007.38-4392.41) | -11.10 | -18.70 | -27.72 |
|  | Syrian Arab Republic | 6587.2(5398.21-7866.53) | 5964.47(5119.58-6829.58) | 5686.87(4555.74-7156.88) | -9.45 | -4.65 | -13.67 |
|  | Egypt | 7225.53(6511.26-8239.16) | 6931.61(6158.29-7702.53) | 6788.35(5140.23-8442.55) | -4.07 | -2.07 | -6.05 |
| Low middle | Djibouti | 1363.77(1064.43-1747.5) | 1621(1164.96-2206.96) | 1697.85(1197.6-2404.8) | 18.86 | 4.74 | 24.50 |
|  | Morocco | 5151.69(4426.4-5991.02) | 4902.89(4103.87-5861.08) | 4322.55(3441.03-5233.09) | -4.83 | -11.84 | -16.09 |
|  | Sudan | 6173.02(4990.63-7545.11) | 4939.7(3632.65-6488.41) | 4409.56(3392.85-5736.27) | -19.98 | -10.73 | -28.57 |
| Low | Somalia | 1577.75(1048.58-2258.79) | 1831.05(1230.79-2630.26) | 1943.96(1359.67-2770.75) | 16.05 | 6.17 | 23.21 |
|  | Pakistan | 2783.77(2232.32-3394.23) | 3385.2(2905.89-3962.9) | 3165.25(2513.87-4040.34) | 21.60 | -6.50 | 13.70 |
|  | Yemen | 5895.01(4707.35-7335.15) | 4884.11(3913.83-6094.94) | 4760.37(3828.5-6181.3) | -17.15 | -2.53 | -19.25 |
|  | Afghanistan | 8452.68(6587.27-10783.97) | 8010.01(6039.78-10504.68) | 6608.19(5054.82-8563.18) | -5.24 | -17.50 | -21.82 |

**^*^**95% uncertainty intervals (UI) gathered from GBD website.
