# Supplementary material for: The burden of ischemic heart disease and the epidemiologic transition in the Eastern Mediterranean Region: 1990–2019
Source: PLoS One. 2023 Sep 5;18(9):e0290286. doi: 10.1371/journal.pone.0290286 (PMC10479892; doi:10.1371/journal.pone.0290286)
Supplement: S9 File — (DOCX) [file pone.0290286.s009.docx]

S9. Comparison of age-standardized DALY attribute risk factors (metabolic, behivioral and enviormental) rate of IHD (per 100,000) in 1990,2005 and 2019 by SDI level and EMR countries.

| SDI  level | Location | Metabolic risks | | | %Δ ($\frac{x_{i+1}-x_{i}}{x_{i}})$ | | | Behavioral risks | | | %Δ ($\frac{x_{i+1}-x_{i}}{x_{i}})$ | | | Environmental risks | | | %Δ ($\frac{x_{i+1}-x_{i}}{x_{i}})$ | | |
| --- | --- | --- | --- | --- | --- | --- | --- | --- | --- | --- | --- | --- | --- | --- | --- | --- | --- | --- | --- |
|  |  | 1990 | 2005 | 2019 | 1990-2005 | 2005-2019 | 1990-2019 | 1990 | 2005 | 2019 | 1990-2005 | 2005-2019 | 1990-2019 | 1990 | 2005 | 2019 | 1990-2005 | 2005-2019 | 1990-2019 |
| - | Global | 2589.126 | 2229.793 | 1881.302 | -13.88 | -15.63 | -27.34 | 2273.665 | 1891.154 | 1538.601 | -16.82 | -18.64 | -32.33 | 1007.944 | 861.9244 | 721.6801 | -14.49 | -16.27 | -28.40 |
|  | EMR | 4423.918 | 4477.889 | 4033.687 | 1.22 | -9.92 | -8.82 | 3949.567 | 3701.438 | 3176.922 | -6.28 | -14.17 | -19.56 | 2287.256 | 2176.716 | 1829.007 | -4.83 | -15.97 | -20.03 |
| High | Kuwait | 3541.005 | 2852.526 | 2031.249 | -19.44 | -28.79 | -42.64 | 2896.864 | 2256.619 | 1580.002 | -22.10 | -29.98 | -45.46 | 1569.127 | 1283.027 | 883.9292 | -18.23 | -31.11 | -43.67 |
|  | United Arab Emirates | 4962.064 | 5073.44 | 3051.381 | 2.24 | -39.86 | -38.51 | 3488.289 | 3311.229 | 2078.036 | -5.08 | -37.24 | -40.43 | 1963.342 | 1968.584 | 1213.007 | 0.27 | -38.38 | -38.22 |
|  | Qatar | 6242.088 | 4885.363 | 3173.398 | -21.74 | -35.04 | -49.16 | 4452.894 | 3223.305 | 2043.672 | -27.61 | -36.60 | -54.10 | 2766.949 | 2110.414 | 1295.368 | -23.73 | -38.62 | -53.18 |
| High  Middle | Libya | 3277.487 | 2807.904 | 3081.422 | -14.33 | 9.74 | -5.98 | 2600.228 | 2134.915 | 2341.352 | -17.90 | 9.67 | -9.96 | 1300.728 | 1066.925 | 1096.979 | -17.97 | 2.82 | -15.66 |
|  | Jordan | 3759.588 | 3032.973 | 2029.996 | -19.33 | -33.07 | -46.00 | 3219.219 | 2419.066 | 1593.782 | -24.86 | -34.12 | -50.49 | 1333.657 | 1087.776 | 687.4858 | -18.44 | -36.80 | -48.45 |
|  | Saudi Arabia | 3980.263 | 4733.616 | 3801.265 | 18.93 | -19.70 | -4.50 | 3291.655 | 3690.824 | 2936.948 | 12.13 | -20.43 | -10.78 | 1898.242 | 2125.086 | 1657.223 | 11.95 | -22.02 | -12.70 |
|  | Lebanon | 5832.523 | 4164.919 | 4023.817 | -28.59 | -3.39 | -31.01 | 4676.31 | 3313.929 | 3208.593 | -29.13 | -3.18 | -31.39 | 2152.731 | 1477.476 | 1331.085 | -31.37 | -9.91 | -38.17 |
|  | Bahrain | 7298.301 | 4261.296 | 2278.408 | -41.61 | -46.53 | -68.78 | 5625.901 | 3069.799 | 1588.833 | -45.43 | -48.24 | -71.76 | 3020.695 | 1732.32 | 876.1565 | -42.65 | -49.42 | -70.99 |
|  | Oman | 7117.47 | 6990.841 | 4832.397 | -1.78 | -30.88 | -32.11 | 6285.979 | 5266.014 | 3379.585 | -16.23 | -35.82 | -46.24 | 3442.942 | 2872.072 | 1828.168 | -16.58 | -36.35 | -46.90 |
| Middle | Tunisia | 3556.105 | 3369.036 | 2922.236 | -5.26 | -13.26 | -17.82 | 2890.849 | 2539.416 | 2088.777 | -12.16 | -17.75 | -27.75 | 1388.525 | 1254.42 | 1036.899 | -9.66 | -17.34 | -25.32 |
|  | Iran (Islamic Republic of) | 4423.986 | 3395.291 | 2438.557 | -23.25 | -28.18 | -44.88 | 3831.004 | 2703.511 | 1855.826 | -29.43 | -31.35 | -51.56 | 2026.008 | 1473.195 | 997.3038 | -27.29 | -32.30 | -50.77 |
|  | Iraq | 5429.485 | 5096.394 | 4326.298 | -6.13 | -15.11 | -20.32 | 4625.986 | 4249.341 | 3435.53 | -8.14 | -19.15 | -25.73 | 2415.343 | 2150.832 | 1720.9 | -10.95 | -19.99 | -28.75 |
|  | Syrian Arab Republic | 6512.774 | 5720.824 | 5653.56 | -12.16 | -1.18 | -13.19 | 5567.931 | 4612.15 | 4407.42 | -17.17 | -4.44 | -20.84 | 2746.65 | 2242.829 | 2115.399 | -18.34 | -5.68 | -22.98 |
|  | Egypt | 6328.133 | 6258.789 | 6116.123 | -1.10 | -2.28 | -3.35 | 5513.334 | 4657.672 | 4383.98 | -15.52 | -5.88 | -20.48 | 3263.348 | 3035.621 | 2819.93 | -6.98 | -7.11 | -13.59 |
| Low  Middle | Djibouti | 1325.899 | 1653.051 | 1727.89 | 24.67 | 4.53 | 30.32 | 1364.562 | 1543.55 | 1502.513 | 13.12 | -2.66 | 10.11 | 757.0448 | 771.6108 | 752.0914 | 1.92 | -2.53 | -0.65 |
|  | Morocco | 5223.792 | 4706.169 | 4598.639 | -9.91 | -2.28 | -11.97 | 4312.623 | 3322.597 | 3112.299 | -22.96 | -6.33 | -27.83 | 2064.437 | 1796.095 | 1679.318 | -13.00 | -6.50 | -18.65 |
|  | Sudan | 6175.413 | 5259.867 | 4698.833 | -14.83 | -10.67 | -23.91 | 5722.496 | 4528.671 | 3824.459 | -20.86 | -15.55 | -33.17 | 3754.536 | 2825.714 | 2242.402 | -24.74 | -20.64 | -40.27 |
| Low | Somalia | 1647.68 | 1837.225 | 1921.983 | 11.50 | 4.61 | 16.65 | 1672.138 | 1708.832 | 1695.713 | 2.19 | -0.77 | 1.41 | 1257.021 | 1312.703 | 1303.719 | 4.43 | -0.68 | 3.71 |
|  | Pakistan | 2501.266 | 3653.427 | 3565.427 | 46.06 | -2.41 | 42.54 | 2468.109 | 3492.424 | 3165.595 | 41.50 | -9.36 | 28.26 | 1490.747 | 2047.785 | 1849.06 | 37.37 | -9.70 | 24.04 |
|  | Yemen | 5800.878 | 4918.629 | 4849.533 | -15.21 | -1.40 | -16.40 | 5844.746 | 4694.574 | 4485.539 | -19.68 | -4.45 | -23.26 | 3696.78 | 2699.176 | 2438.117 | -26.99 | -9.67 | -34.05 |
|  | Afghanistan | 7523.398 | 7199.549 | 5932.397 | -4.30 | -17.60 | -21.15 | 6771.28 | 6372.195 | 5041.664 | -5.89 | -20.88 | -25.54 | 4991.776 | 4666.675 | 3365.485 | -6.51 | -27.88 | -32.58 |
