# Supplementary material for: The burden of ischemic heart disease and the epidemiologic transition in the Eastern Mediterranean Region: 1990–2019
Source: PLoS One. 2023 Sep 5;18(9):e0290286. doi: 10.1371/journal.pone.0290286 (PMC10479892; doi:10.1371/journal.pone.0290286)
Supplement: S10 File — (DOCX) [file pone.0290286.s010.docx]

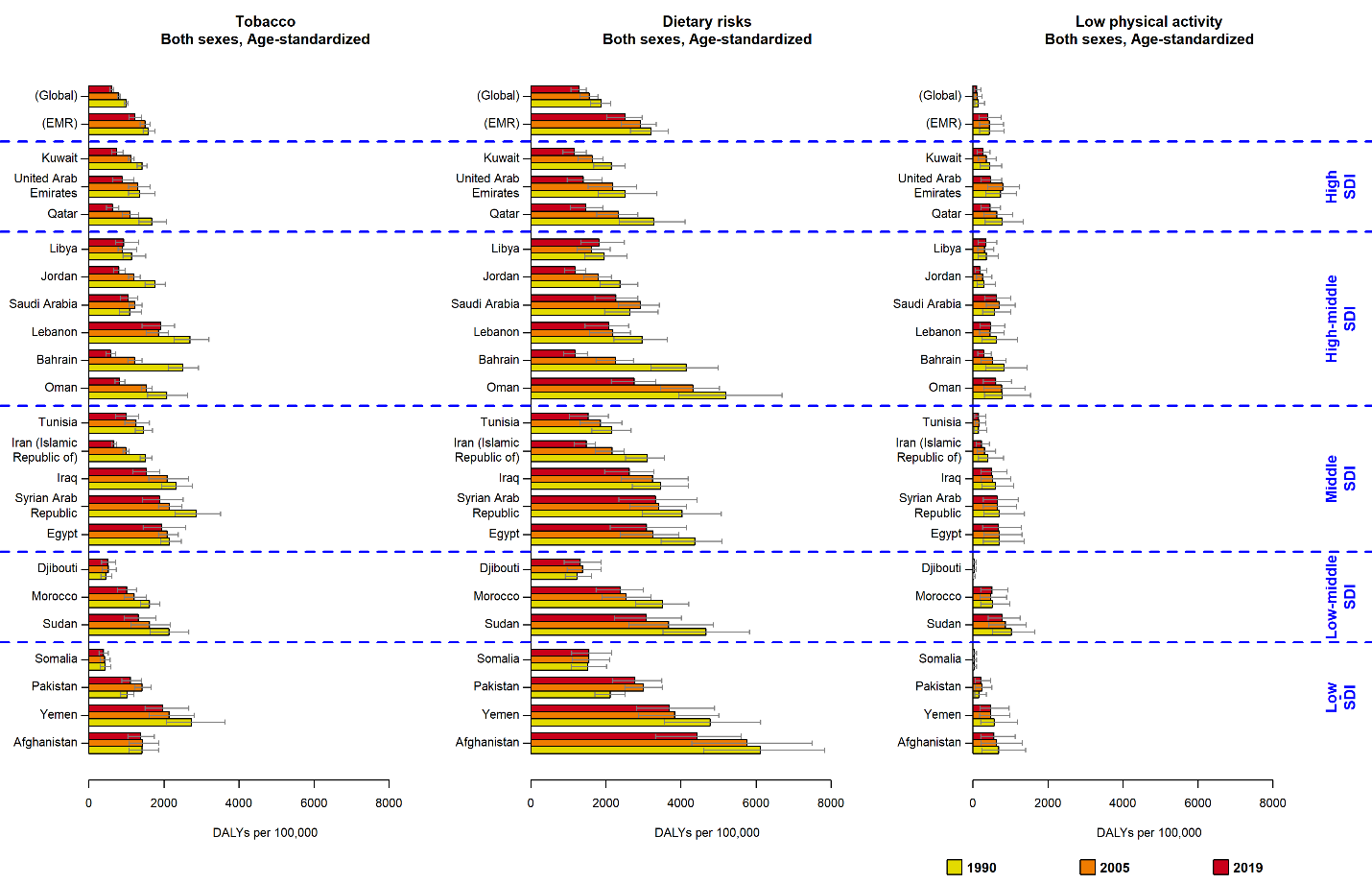


S10. Comparison of three DALYs attributed behavioral risk factors tobacco, dietary risk and low physical activity by SDI level and geographical area.
