# Supplementary material for: The burden of ischemic heart disease and the epidemiologic transition in the Eastern Mediterranean Region: 1990–2019
Source: PLoS One. 2023 Sep 5;18(9):e0290286. doi: 10.1371/journal.pone.0290286 (PMC10479892; doi:10.1371/journal.pone.0290286)
Supplement: S11 File — (DOCX) [file pone.0290286.s011.docx]

S11. Comparison of disability-adjusted life years (DALYs) attribute risk factors in global and EMR countries in 3-point time 1990,2005 and 2019.

| **Risk factors** | **1990(95%UI)** | | **2005(95%UI)** | | **2019(95%UI)** | |
| --- | --- | --- | --- | --- | --- | --- |
|  | **Global** | **EMR** | **Global** | **EMR** | **Global** | **EMR** |
| **High systolic blood pressure** | 1,710.85  (1,465.77-1,965.39) | 2,809.81  (2,351.72-3,286.23) | 1,458.27  (1,247.48-1,679.71) | 2,846.68  (2,393.65-3,315.79) | 1,217.9  (1,032.03-1,412.44) | 2,618.04  (2,140.39-3,141.3) |
| **High LDL Cholesterol** | 1,530.7  (1,263.94-1,819.61) | 2,516.03  (2,049.96-3,015.43) | 1,260.42  (1,034.59-1,495.84) | 2,486.46  (2,034.12-2,954.44) | 1,036.94  (843.78-1,244.99) | 2,188.37  (1,738.96-2,694.59) |
| **Particulate matter pollution** | 872.16  (833.13-912.46) | 1,778.82  (1,533.01-2,054.97) | 647.43  (572.75-725.8) | 1,657.92  (1,467.92-1,870.88) | 547.82  (478.99-618.84) | 1,379.05  (1,167.83-1,608.56) |
| **Smoking** | 766.51  (663.72-869.35) | 1,298.34  (1,185.29-1,430.73) | 695.7  (664.58-728.25) | 1,225.17  (1,131.26-1,341.05) | 519.48  (481.37-559.19) | 998.41  (868.63-1,144.84) |
| **Diet low in whole grains** | 568.15  (215.21-730.62) | 1,254.25  (513.2-1,571.64) | 486.21  (183.5-626.6) | 1,205.5  (496.06-1,512.33) | 393.56  (148.89-511.19) | 1,038.59  (423.66-1,332.98) |
| **High BMI** | 521.77  (289.4-810.94) | 1,069.77  (627.24-1,627.15) | 525.48  (318.29-772.78) | 1,257.51  (783.3-1,795.43) | 499.41  (313.3-709.95) | 1,319.95  (846.63-1,855.14) |
| **High fasting plasma glucose** | 534.79  (331.65-804.82) | 837.75  (546.66-1,267.58) | 547.14  (351.78-823.32) | 1,174.54  (741.37-1,776.44) | 534.84  (340.7-792.23) | 1,374.18  (843.87-2,085.46) |

**^*^**95% uncertainty intervals (UI) gathered from GBD website.
