# Supplementary figures and images for: The burden of ischemic heart disease and the epidemiologic transition in the Eastern Mediterranean Region: 1990–2019
Source: PLoS One. 2023 Sep 5;18(9):e0290286. doi: 10.1371/journal.pone.0290286 (PMC10479892; doi:10.1371/journal.pone.0290286)

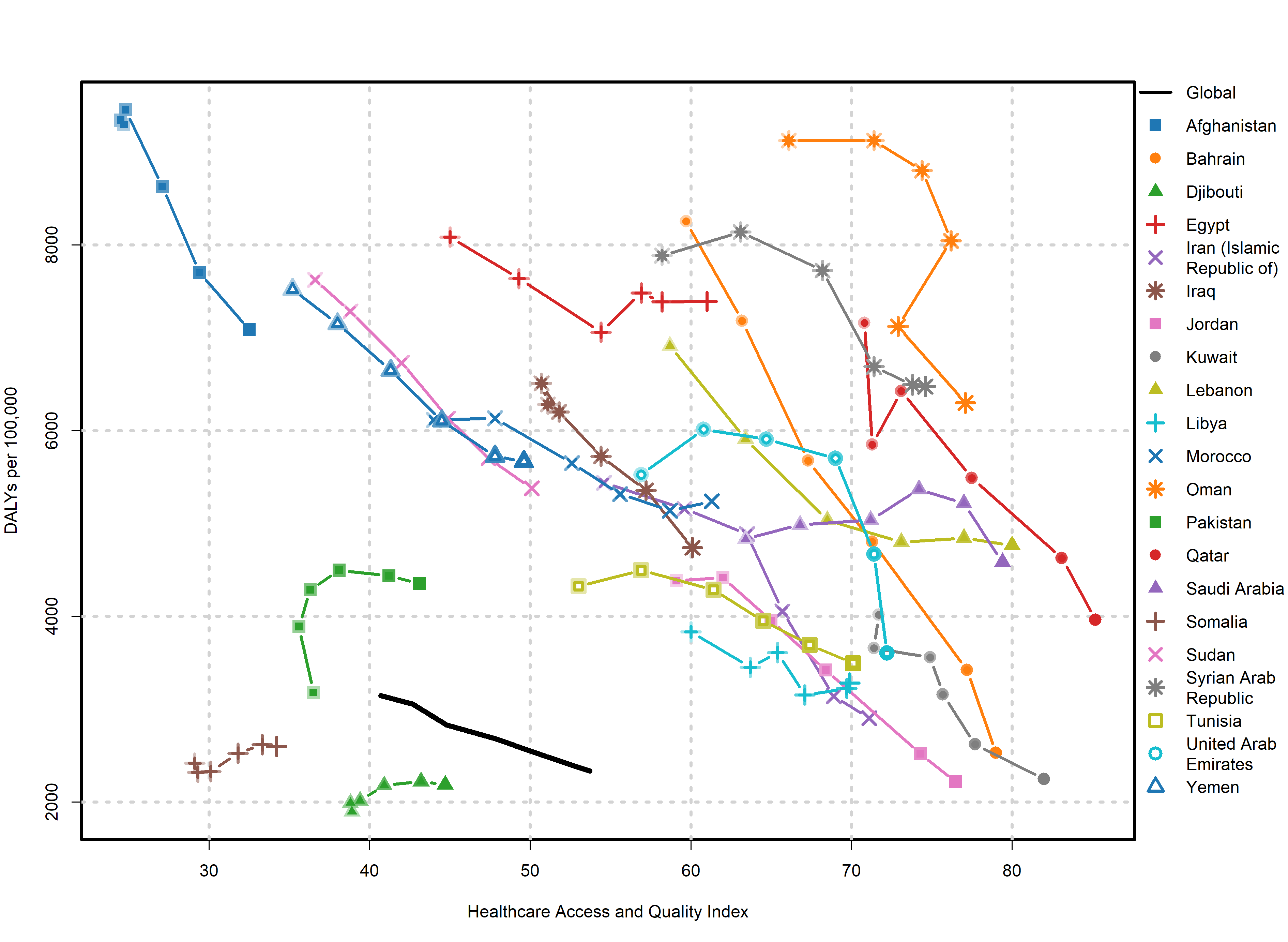


S12. The relationship healthcare access and quality index (HAQ) and DALY by EMR countries

Supplement: S12 File — (DOCX) [file pone.0290286.s012.docx]
